# Disulfide driven folding for a conditionally disordered protein

**DOI:** 10.1038/s41598-017-17259-4

**Published:** 2017-12-05

**Authors:** Hugo Fraga, Jordi Pujols, Marcos Gil-Garcia, Alicia Roque, Ganeko Bernardo-Seisdedos, Carlo Santambrogio, Joan-Josep Bech-Serra, Francesc Canals, Pau Bernadó, Rita Grandori, Oscar Millet, Salvador Ventura

**Affiliations:** 1 Institut de Biotecnologia i Biomedicina. Universitat Autònoma de Barcelona, 08193 Bellaterra, Spain; 2 Departament de Bioquímica i Biologia Molecular, Universitat Autònoma de Barcelona, 08193 Bellaterra, Spain; 3 Departamento de Bioquimica, Faculdade de Medicina da Universidade do Porto, Porto, Portugal; 4 Protein Stability and Inherited Diseases Laboratory, CIC bioGUNE, 48160 Derio, Spain; 5 Department of Biotechnology and Biosciences, University of Milano-Bicocca, Milano, Italy; 6 Vall d’Hebron Institute of Oncology (VHIO), Barcelona, Spain; 7 Centre de Biochimie Structurale, INSERM-U1054, CNRS UMR-5048, Université de Montpellier, 29, rue de Navacelles, 34090 Montpellier, France

## Abstract

Conditionally disordered proteins are either ordered or disordered depending on the environmental context. The substrates of the mitochondrial intermembrane space (IMS) oxidoreductase Mia40 are synthesized on cytosolic ribosomes and diffuse as intrinsically disordered proteins to the IMS, where they fold into their functional conformations; behaving thus as conditionally disordered proteins. It is not clear how the sequences of these polypeptides encode at the same time for their ability to adopt a folded structure and to remain unfolded. Here we characterize the disorder-to-order transition of a Mia40 substrate, the human small copper chaperone Cox17. Using an integrated real-time approach, including chromatography, fluorescence, CD, FTIR, SAXS, NMR, and MS analysis, we demonstrate that in this mitochondrial protein, the conformational switch between disordered and folded states is controlled by the formation of a single disulfide bond, both in the presence and in the absence of Mia40. We provide molecular details on how the folding of a conditionally disordered protein is tightly regulated in time and space, in such a way that the same sequence is competent for protein translocation and activity.

## Introduction

Deciphering how an unstructured polypeptide folds into its biologically active three-dimensional structure has been one of the major challenges in biochemistry over the last 50 years^1^. A first problem in folding studies is that the biological unfolded state cannot be directly accessed, but only approximated, i.e. using strongly denaturing conditions^2^. A second limitation is that due the high speed at which proteins generally fold, most experiments measure the rate of appearance or disappearance of a specific signature for a particular species, typically on millisecond-second scales^3,4^. Spectroscopic probes like fluorescence^5^ and circular dichroism (CD)^6^ are usually used in these assays, since they display enough intrinsic time resolution to follow folding reactions. These data provide important information on the height of energetic barriers, but say little about how protein conformation evolves along the pathway. Accordingly, alternative techniques like Nuclear Magnetic Resonance (NMR)^7^, infrared spectroscopy (FTIR)^8^, small-angle X-ray scattering (SAXS)^9^ or native mass spectrometry (MS)^10^ have been implemented to track the folding of certain proteins, providing novel and wealthy structural information in these specific cases. Because no single method can provide a entire picture of the folding reaction, the integration of all those complementary approaches to characterize the complete folding trajectory of a given polypeptide is a long pursued objective.

A large number of cysteine-containing proteins encoded in the nucleus and synthesized on cytosolic ribosomes are targeted to the intermembrane space (IMS) of mitochondria as reduced species and they become oxidized into their functional forms only after entering this organelle compartment^11^. The IMS oxidoreductase Mia40 introduces disulfide bonds into these target proteins to facilitate their folding^11,12^.

The unique properties of Mia40 substrates offer an opportunity to characterize folding reactions with unprecedented conformational detail. First, a biologically relevant unfolded state can be accessed, since in their reduced state these proteins remain essentially unstructured, both *in vitro* and inside cells^13^. Second, non-catalyzed folding of these proteins can take several hours to occur^14^ which permits tracking the process in real-time by a battery of low temporal-resolution techniques reporting on different structural properties. Third, in these proteins the adoption of the native structure and the formation of the disulfide bonds are linked in a process known as oxidative folding, allowing an accurate characterization of the major transient intermediates that populate the folding reaction^15^.

**Figure 1:**
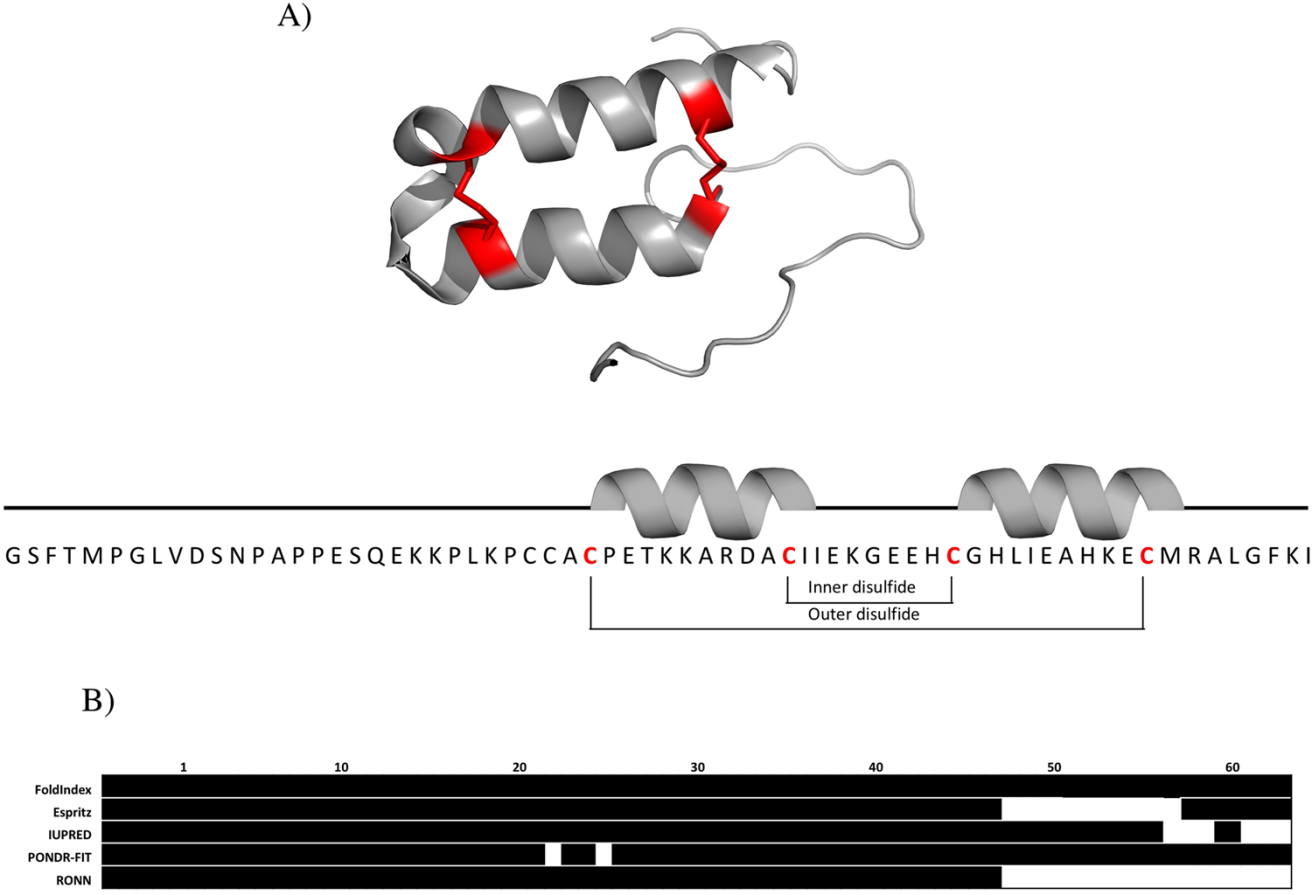
hCox17 structure and disorder prediction. (**A**) Upper, 3D representation of hcox17 (pdb code: 2R9N) with red-colored cysteines and disulfides. Bottom, primary sequence and secondary structure of hCox17. (**B**) Disorder predictions for hCox17 (C26S, C36S, C45S, C55S) sequence using Foldindex, Espritz, IUpred, Pondr-Fit and Ronn. Those amino acids above the methodology threshold and predicted as disordered are colored in white.

Cox17 is one of the best-characterized substrates of Mia40. It acts in the IMS as the donor of Cu(I) to both Sco1 and Cox11^16,17^, being also involved in the regulation of mitochondrial contact sites and cristae organization^18^. Mutations in Cox17 result in the dysfunction of cytochrome C oxidase, leading to respiratory defects^19^. Human Cox17 (hCox17) belongs to the CX_9_C family and is a 62-residue protein containing six conserved Cys. The NMR solution structure of this protein shows that it consists of a coiled-coil-helix-coiled-coil-helix domain (CHCH) stabilized by two disulfide bonds involving Cys26–Cys55 and Cys36–Cys45, preceded by a flexible and completely unstructured N-terminal tail **(**Fig. 1A **)**. The Cu(I) ion is coordinated by two additional free cysteines at positions 23 and 24.

The folding of hCox17 can be considered as a disorder-to-order transition mediated by a post-translational modification: the formation of structural disulfide bonds^20,21^. In the present study, we integrate real-time chromatography, disulfide, fluorescence, CD, FTIR, SAXS, MS and NMR analyses in order to provide a complete conformational description of the mechanism governing this structural switch.

## Results

### hCox17 is predicted as an IDP in the reduced state

We have recently proposed that many small proteins containing disulfide bonds would resemble bona fide IDPs, when in their reduced states^22^. Despite there is no perfect mimic for reduced Cys residues, mutation of disulfide-forming Cys to Ser is considered the less disruptive change to simulate disulfide bond reduction^23^. We virtually mutated Cys to Ser in hCox17 sequence to mimic the reduced state of the protein and analyzed the degree of predicted disorder using FoldIndex^24^, Espritz^25^, IUPRED^26^, PONDR-FIT^27^ and RONN^28^ algorithms. With the exception of a small stretch at the hCox17 C-terminus, all these programs consistently predict this sequence as mostly disordered (Fig. 1B).

### Reduced hCox17 is mostly disordered

Previous folding studies on yeast Cox17 (yCox17), which has only 35% sequence identity with hCox17, have employed mutants where several Cys were changed to Ser^29^. In order to attain a comprehensive view of the complexity of hCox17 folding pathway we used here instead the human WT protein, where all 6 Cys are present. For example, reducing this number to 4 Cys would decrease the potential disulfide isomers in the folding pathway from 75 (15 1S-S + 45 2S-S + 15 3S-S) to only 9 (6 1S-S + 3 2S-S).

**Figure 2:**
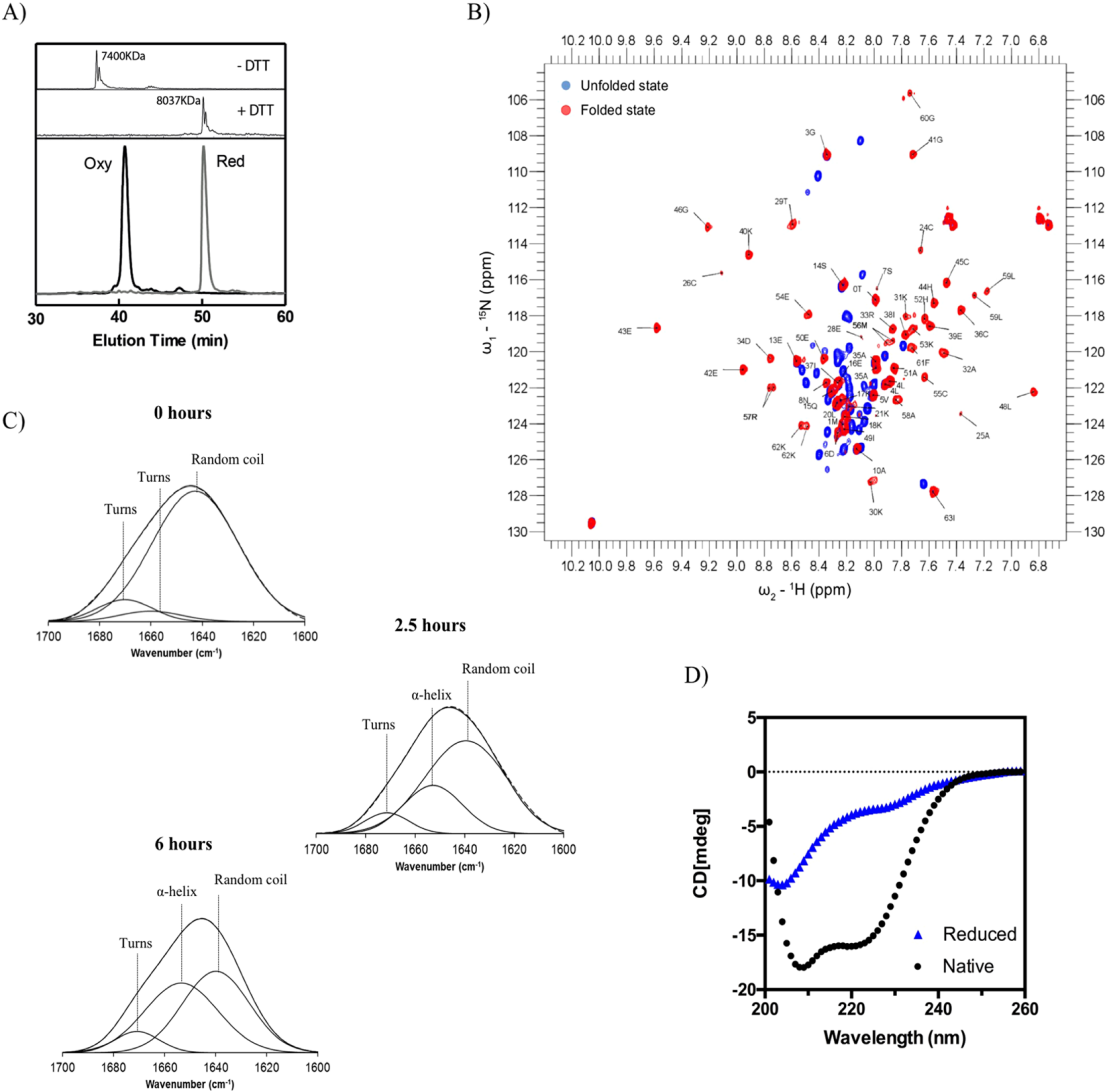
Conformational properties of reduced and native hCox17. (**A**) Lower panel: HPLC chromatograms of native (black) and 2 hours DTT-treated hCox17 (grey). The upper panel shows MALDI-TOF spectra of both samples quenched with vinylpiridine. (**B**) NMR ^1^H-^15^N-HSQC spectrum of reduced (blue) and native (red) hCox17 with the respective assignments. (**C**) FTIR absorbance spectra of hCox17 in the amide region at several time points of the refolding reaction. Experimental spectra and their fitting are shown in dashed and solid lines, respectively. (**D**) Far-UV CD spectra of reduced (blue triangles) and native (black circles) hCox17.

It has been suggested that hCox17 is significantly unfolded when reduced and that this is probably pivotal for its diffusion across the mitochondrial outer membrane^30^. In order to confirm this point, hCox17 purified from *E.coli* Origami cells was reduced with 200 mM DTT for 2 hours. hCox17 reduction was clear from the shift in RP-HPLC retention time (Fig. 2A). The oxidation state of the protein was confirmed using MALDI-TOF after reaction with vinylpiridine. An increase of 637 Da (105 of vynilpiridine per free SH + 1 proton per SH) was observed when native hCox17 was reduced and treated with vinylpiridine indicating that all 6 Cys were accessible in the reduced state.

**Figure 3:**
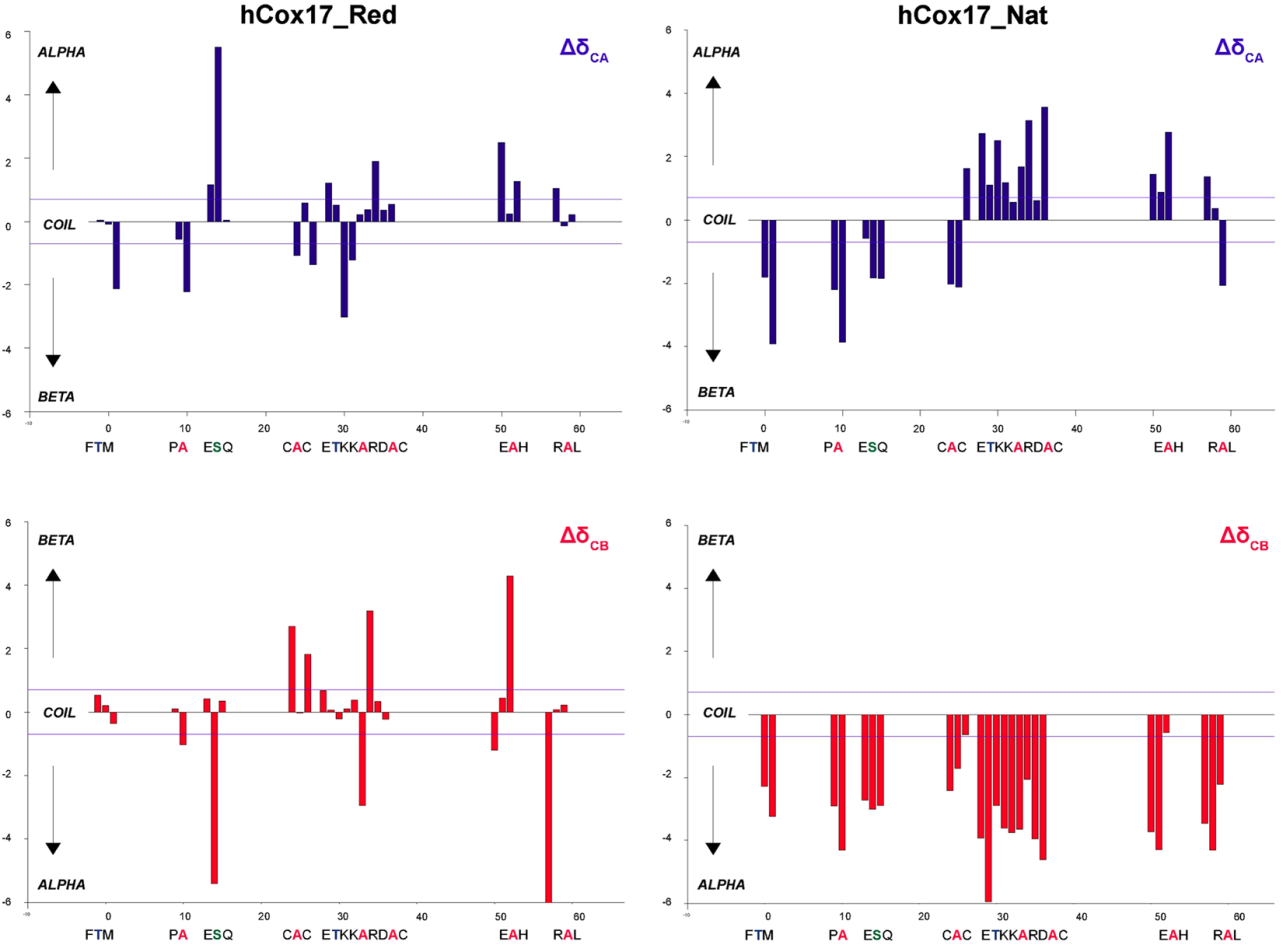
Random Coil Index values for Cα and Cβ chemical shifts. Values above 0.7 in Cα are indicative of α-helix whereas values below −0.7 are indicative of β-sheet and vice versa for those values of Cβ.

The NMR ^1^H-^15^N-HSQC spectrum of reduced hCox17 (Fig. 2B) is consistent with an IDP fingerprint: The amide groups of hCox17 protein are in a similar chemical environment which leads to a clustering of 41 15N - 1H distinguishable signals in the central part of the spectrum, with strong peak intensities and a characteristic up-field position for the ε–TRP signal. For folded hCox17, the chemical environments are more heterogeneous due to the presence of the two alpha-helices, thus creating a more disperse spectrum (Fig. 2B). We further investigated the structural properties of reduced hCox17 by recording the ^1^H-^15^N-HSQC and HNCACB spectra of the ^15^N-^13^C-labelled protein. Chemical shifts were assigned for Ala, Trp and Ser and their neighboring residues (Fig. S1), as they fall in isolated Cβ chemical shift regions. Reference chemical shift values for the same residues in the oxidized state were obtained from BMRB 11019 (Table S1). Protein flexibility and secondary structure was predicted by comparing the Random Coil index values from the backbone chemical shifts (HN, N, Cα and Cβ)^31^ (Fig. 3). Based on the analyzed residues it can be deduced that reduction of hCox17 results in the loss of most of its secondary structure content.

These results agree with the Fourier transform infrared spectroscopy (FTIR) spectrum of DTT treated hCox17 in the amide I region, which evidences the lack of significant regular secondary structure (Fig. 2C), whereas the FTIR of spectrum of native hCox17 contains a 44% α-helical content (Fig. 2C and Table S2). This percentage of α-helix is in perfect agreement with the solution structure of hCox17 (30/67 residues in α-helix = 44%).

The far-UV circular dichroism (CD) of native hCox17 is typical of an α-helix containing protein with characteristic minima at ≈210 nm and ≈222 nm (Fig. 2D). The far-UV CD spectra of reduced hCox17 displays much lower ellipticity and a minimum at 205 nm consistent with a mostly disordered structure (Fig. 2D). However, a shoulder at ≈222 nm was evident in the spectrum. To discard that this signal could be indicative of a certain residual α-helical content, we performed a thermal denaturation of reduced hCox17 and monitored the changes in α-helix content following the changes in the CD signal at 222 nm. We did not observe any change in ellipticity along the melting (Fig. S2A) and the CD spectra of the reduced protein at 25 and 95 °C were highly similar (Fig. S2B), in contrast to what happens with the oxidized protein (Fig. S2C). This lack of structural transition is in agreement with the NMR and FTIR data, supporting hCox17 being mostly disordered in the reduced state.

The disordered N-terminus of native hCox17 accounts for roughly 50% of its sequence, this affects the hydrodynamic properties of the protein. Accordingly, native hCox17 elutes much earlier than expected for its molecular weight in a size exclusion chromatography (SEC) (apparent MW ≈14.000 Da/predicted MW = 7.400). Reduction of hCox17 further increases its hydrodynamic volume and the protein elutes earlier than the native form (apparent MW ≈20.500 Da), which is consistent with a larger fraction of the protein populating disordered conformations (Fig. S3).

### Cox17 folding proceeds by sequential disulfide formation

To monitor hCox17 folding, we first analyzed the progression of hCox17 secondary structure formation using CD. The reduced protein was loaded in a PD10 desalting column (GE Healthcare) to remove DTT, allowing the protein to re-oxidize its free thiol groups into disulfides as previously described^14^. As the reaction progresses the ellipticity of the protein increases. By plotting the changes in ellipticity at 222 nm versus time it can be observed that hCox17 folding is extremely slow, taking ≈16 h for completion at pH 7.0 (Fig. S4).

To further characterize hCox17 folding, we used FTIR spectroscopy coupled to RP-HPLC analysis. Time-resolved FTIR has proven to be a powerful technique to follow conformational changes during protein folding^8^. On the other hand, RP-HPLC, after quenching the folding reaction with acid, allows the resolution of the different isomers that populate the pathway^14^.

**Figure 4:**
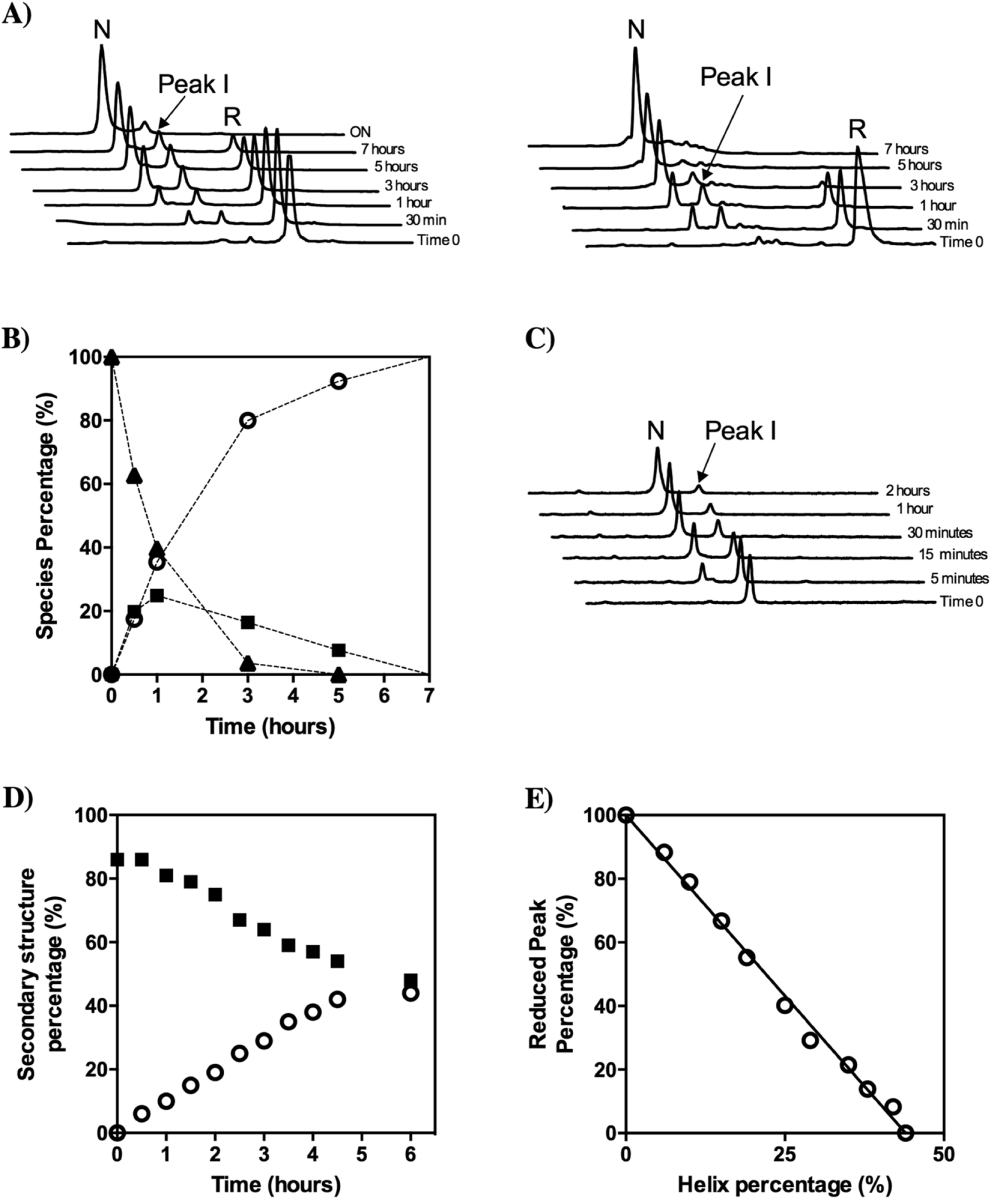
Inner disulfide bond species represents a folding intermediate and displays secondary structure features linked with the native conformation. (**A**) hCox17 was allowed to re-oxidize after DTT removal at pH 7 (left) and pH 8.4 (right). At defined time points, the reaction was quenched and analyzed by HPLC. (**B**) RP-HPLC peak areas, from the refolding reaction at pH 8.4, are plotted as function of time. Reduced (solid triangles), intermediate (solid squares) and native (open circles) species. (**C**) Acid-trapped intermediate I was purified by RP-HPLC, freeze-dried, and dissolved in Tris-HCl (pH 8.4) to reinitiate the folding reaction. Folding reaction was subsequently quenched with acid at different time points and re-analyzed by RP-HPLC. (**D**) Reduced hCox17 was allowed to air oxidize at pH 8.4 inside a sealed FTIR spectrometer cuvette and the spectra recorded at specific refolding times. The relative contributions of random coil (solid squares) and α-helix (open circles) conformations to the global amide I spectra are plotted as a function of time. (**E**) Duplicates from the FTIR assay at pH 8.4 were acid-quenched and the different species present in the reaction analyzed by RP-HPLC as indicated above. The area of the reduced specie peak is plotted versus the α-helix content as deduced by FTIR.

hCox17 oxidative folding chromatograms are rather simple, considering the high number of potential disulfide isomers, 75, for a protein containing 6 Cys residues. Apart from the starting fully reduced form (peak R) we could only detect two major species, peak I and peak N (Fig. 4A), displaying decreasing hydrophobic surface accessible to solvent under chromatographic conditions, according to RP-HPLC retention times. Peak N corresponds to native hCox17, whereas, Peak I contained only the inner C36-C45 native disulfide (Table S3). Therefore, no off-pathway intermediates are populated significantly along the folding reaction. The pathway involves exactly the same species independently of whether the reaction is performed at pH 7.0 or at pH 8.4, where the thiols are more reactive (Cys side chain pKa = 8.37) and the reaction proceeds faster, taking only about 6 h to complete (Fig. 4A). This provides us a mean to match the reaction kinetics with the time scale of the different techniques used in this study, just by controlling the pH of the reaction. MALDI- TOF analysis of the oxidative folding mixtures excluded the formation of any sulfenic or sulfonic species.

The quantitative analysis of disulfide species along the reaction (Fig. 4B) indicates that peak I reaches a steady level before the formation of the native protein (Peak N) is complete; suggesting that the transition between the disordered and native states of hCox17 proceeds via the sequential oxidation of single disulfide bonds, in which the formation of the inner disulfide precedes the formation of the outer one. In order to confirm this point, we performed a stop/go experiment. The inner disulfide acid-trapped intermediate was isolated from oxidative folding mixtures, dried and dissolved in standard buffer at pH 8.4 to resume folding. Peak I leads directly and efficiently to the formation of peak N, without the accumulation of any other alternative species (Fig. 4C).

Consistent with CD data, the analysis of the folding reaction using FTIR indicates an increase in α-helical structure as hCox17 folds. This technique allowed us to confirm that this increase in structure comes at expenses of a decrease in disorder (Fig. 4D). Analyzing the FTIR samples using RP-HPLC we find a complete correlation between the gain in secondary structure and the disappearance of the reduced protein (r^2^ = 0.995) (Fig. 4E), which confirms its disordered nature and indicates that the inner disulfide intermediate already contains a significant degree of α-helical structure.

### Formation of the inner disulfide bond involves a transition between a disordered and a compact native-like state in hCox17

**Figure 5:**
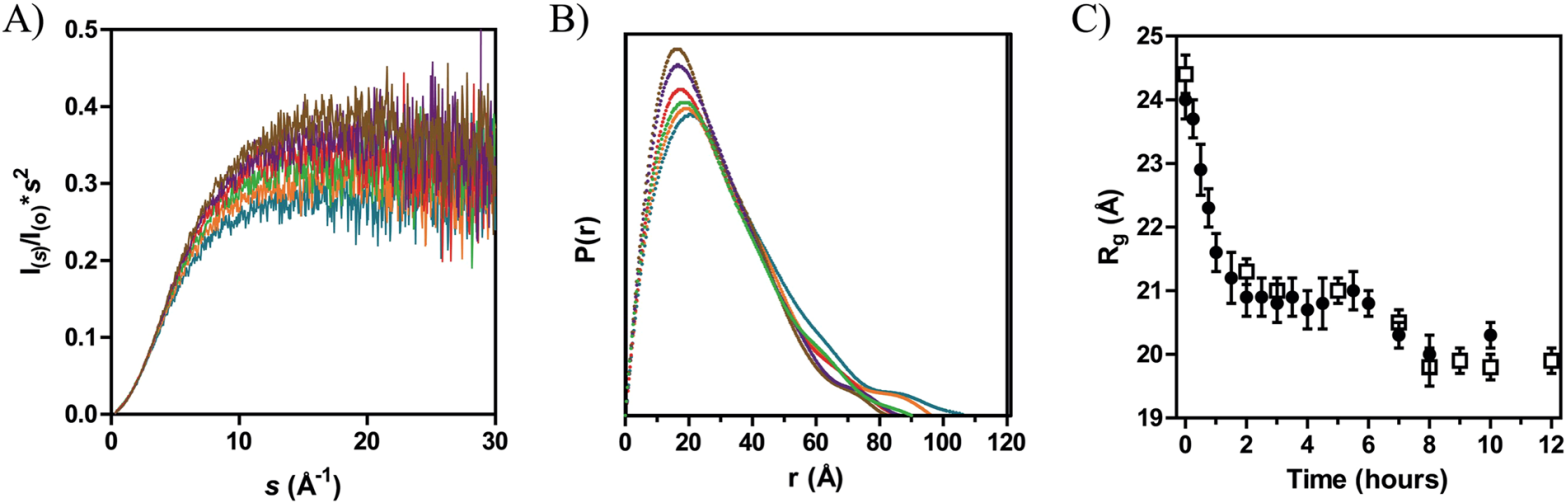
Small-angle X-ray scattering analysis of hCox17 compaction upon folding. (**A**) Kratky representation at selected folding time-points: 0 hours (blue), 30 minutes (orange), 1 hour (green), 2 hours (red), 4 hours (purple) and 8 hours (brown). (**B**) *P*(r) functions representing the time-points colored as in A. (**C**) Radius of Gyration plot versus time of folding. Both 2 mg/mL (spheres) and 3 mg/mL (squares) samples are represented with the error bars. The folding reaction was performed at pH 8.4.

We proceeded testing whether, as suggested by SEC, hCox17 folding resulted in significant changes in protein shape. SAXS can be used for the characterization at low-resolution of protein structure and dynamics^32^. In the context of hCox17, SAXS is useful to assess the time-dependent compaction along the folding pathway. Kratky representations of the measured curves present a continuous increase of the I(*s*)*s* ^2^ with the momentum transfer indicating that reduced hCox17 remains highly disordered (Fig. 5A). Interestingly, the increase in the intensity of the maximum suggests an enhanced compaction with time. This compaction is better monitored by the *P*(r) function that presents more noticeable changes as folding proceeds. Both the systematic decrease of the Dmax value and the shift of the maximum towards smaller distance values demonstrate the compaction process induced by the folding of hCox17 (Fig. 5B). Figure 5C displays the *R*_*g*_ derived from the SAXS curves measured along the oxidative folding of hCox17 at 3 mg/mL and 2 mg/mL at pH 8.4, to check whether protein concentration can increase or disturb global compactation and shape. At initial state, hCox17 exhibits a *R*_*g*_ of 24.2+/−0.3 Å, which is in very good agreement with a fully disordered protein of 67 residues, 22.8 Å^33^. After two hours of reaction hCox17 experiences a fast compaction process to form a particle with an *R*_*g*_ of 21 Å that remains unchanged during the following four hours. Finally, *R*_*g*_ displays a slight decrease of 1 Å after six hours, that remains stable until the end of the assay. A RP-HPLC analysis of the folding reaction under the same conditions than those used in the SAXS experiment (Fig. S5) evidences the presence of around 10% of intermediate at 6 h, suggesting that this last decrease in *R*_*g*_ might reflect its conversion into native hCox17.

**Figure 6:**
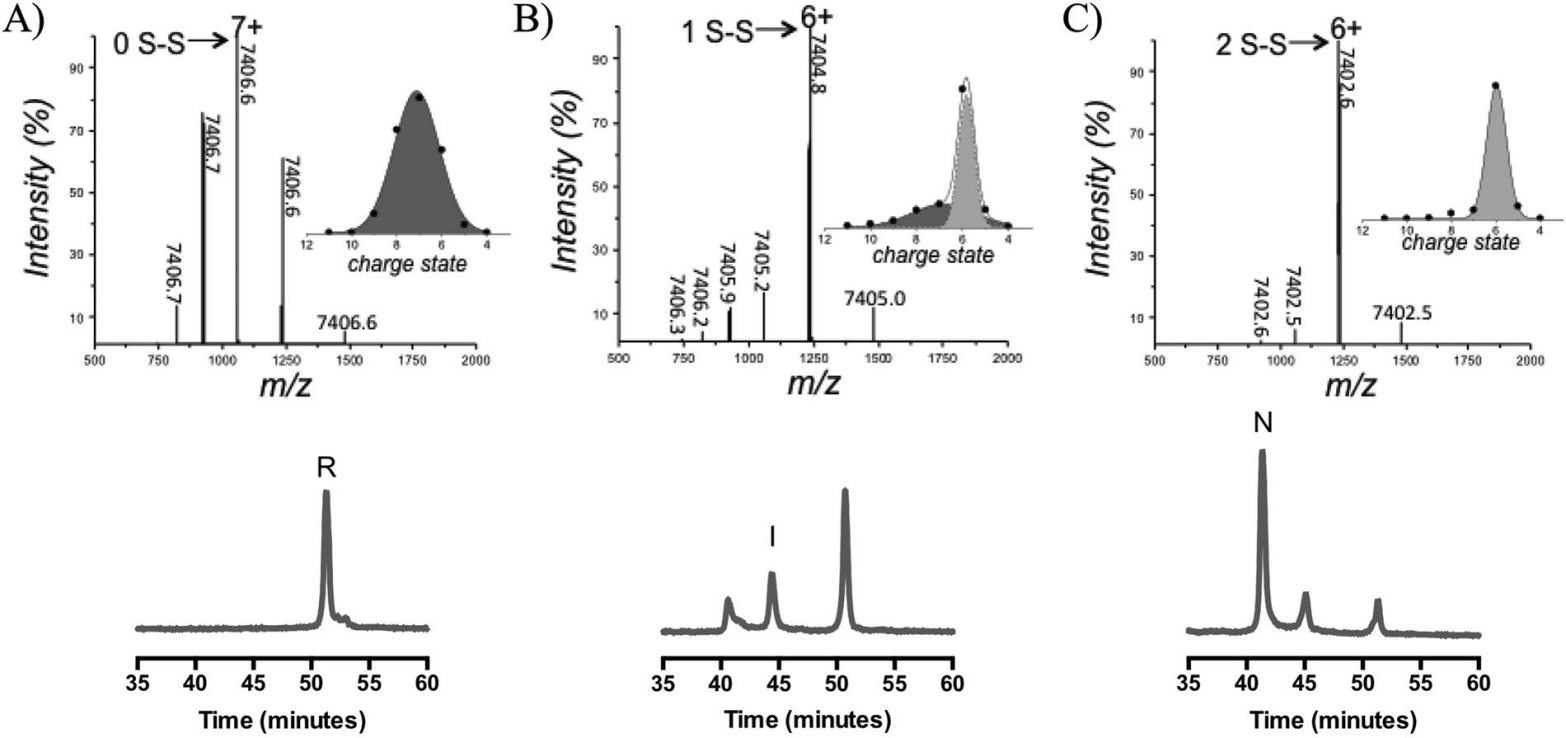
Analysis of the conformation and oxidative state of hCox17 species by native MS. Nano-ESI-MS spectra of 10 μM hCox17 refolding reaction at pH 6.7. hCox17 species are labeled according to its charge state and number of disulfide bridges. Each peak is labeled by the isotopically-averaged mass of the protein. Insets show the Gaussian fitting of the CSD. (**A**) Fully reduced hCox17 in the presence of 50 mM DTT. (**B**) Partially oxidized hCox17, 8 h after the removal of DTT. (**C**) Completely oxidized hCox17, 17 h after the removal of DTT. RP-HPLC analysis is plotted below each time-point, where native (N), intermediate (I) and reduced (R) are labelled.

Comparison of RP-HPLC and SAXS kinetics suggest that formation of the first disulfide and compaction of hCox17 state are connected. To confirm this hypothesis, we used mass spectrometry (MS) under non-denaturing conditions (native MS), which allows monitoring compaction and oxidation distinctly and simultaneously. While accurate mass determination reports on the oxidation state, charge-state distributions (CSDs) allow detection of coexisting conformers^34^. Starting from a fully reduced state, hCox17 folding was initiated at pH 6.7 and samples analyzed by ESI-MS as the reaction proceeds. The fully reduced protein (Fig. 6A) exhibits a broad CSD centered on the 7+ ion and an average mass of 7406.6 (±0.1) Da, (expected 7406.6 Da). An average charge state of 7.2 is in good agreement with the value expected for a completely unstructured protein of this size^35^, confirming the identification of reduced hCox17 as an IDP. Under the conditions employed here, the species containing one disulfide bond attains its maximal accumulation after 8 h (Fig. 6B), as indicated by a mass of 7404.8 (±0.3) Da for the 6+ ion. The mass of the other peaks (7405.0–7406.3 Da) are consistent with mixtures of 1 S-S and fully reduced protein, which indeed still constitutes the major hCox17 specie at this time point in these conditions. Gaussian fitting of the CSD highlights the presence of two conformational components, a narrow one centered on the 6+ ion, corresponding to the 1 S-S intermediate, and a broad one centered on the 7+ ion, corresponding to the fully reduced protein. This result indicates that the formation of the first disulfide bridge is linked to a significant chain compaction, accompanied by a narrowing of the CSD and a shift in average charge state from 7.2 to 5.9. After 17 h of incubation (Fig. 6C), the native 2 S-S form accumulates, as indicated by a further mass shift to 7402.6 (±0.1) Da. However, the prevalent charge state remains 6+ , suggesting that the overall protein compactness is not affected significantly by the formation of the second disulfide bridge.

NMR relaxation data of the amide group can also be used to estimate the rotational diffusion tensor which, in turn, can be reinterpreted in terms of a single correlation time (τ_*x*_) to describe the overall molecular tumbling^36^. Taking advantage of the slow timescale for the folding event, three NMR subsets of signals were independently analyzed to obtain the correlation times for the different species detectable in the NMR spectrum (see below). The extended conformation of the unfolded state results in a τ_*x*_ of 5.9 ± 0.9 ns, while the intermediate form (associated to the formation of the Cys36-Cys45 disulfide bond) shows increased compaction (τ_*x*_ of 6.6 ± 1.3 ns) and, as expected, both species are still more flexible than the native form (τ_*x*_ of 7.1 ± 0.9 ns).

**Figure 7:**
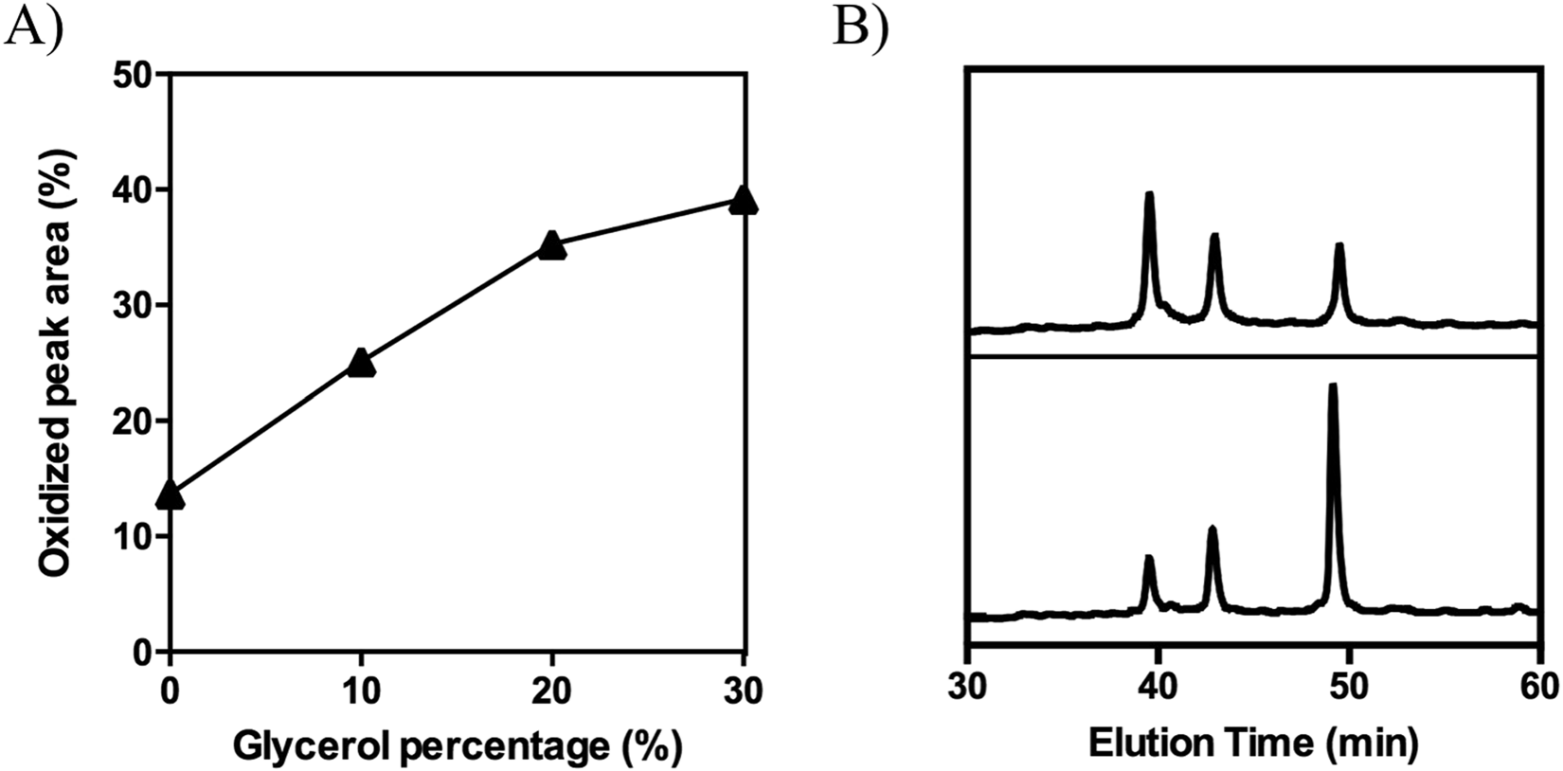
Impact of glycerol on hCox17 folding rates. (**A**) hCox17 was allowed to refold in presence of increasing glycerol concentrations at pH 8.4. After 1 hour of reaction, the mixtures were acid-quenched and analyzed by RP-HPLC. The native peak area is plotted as a function of the glycerol percentage (v/v). (**B**) Representative chromatograms after 1 h of refolding in the absence (bottom) and in the presence (up) of 30% of glycerol. Peaks from left to right: native, intermediate and reduced hCox17.

Overall SAXS, MS and NMR data converge to indicate that the formation of the inner disulfide bond is concomitant with compaction of hCox17. Glycerol is a small polyol know to promote protein compaction, leading to protein conformations with reduced solvent accessible surface area^37^. As it can be seen in Fig. 7A and B, we observed a concentration-dependent increase of folding rates in the presence of glycerol, which further supports compaction as an important event in hCox17 folding.

### Adoption of native structure around the hCox17 inner disulfide precedes outer disulfide formation

In order to collect structural information of the hCox17 folding pathway at the residue level, the reaction was monitored using NMR spectroscopy. For this purpose, ^15^N labelled hCox17 was first reduced in the presence of 100 mM DTT and, following buffer exchange, its oxidation tracked in real time at pH 6.5. The folding reaction progression was monitored by the chemical shift changes in a ^1^H-^15^N-HSQC over a total period of 36 h. In Fig. S6 representative spectra at 0 h, 8 h and 30 h are shown. It can be observed how some signals from the unfolded/reduced state decrease, how signals from the native state arise and how some intermediate signals appears and disappears in the final folded state; thus suggesting that R, I and N are in slow exchange.

**Figure 8:**
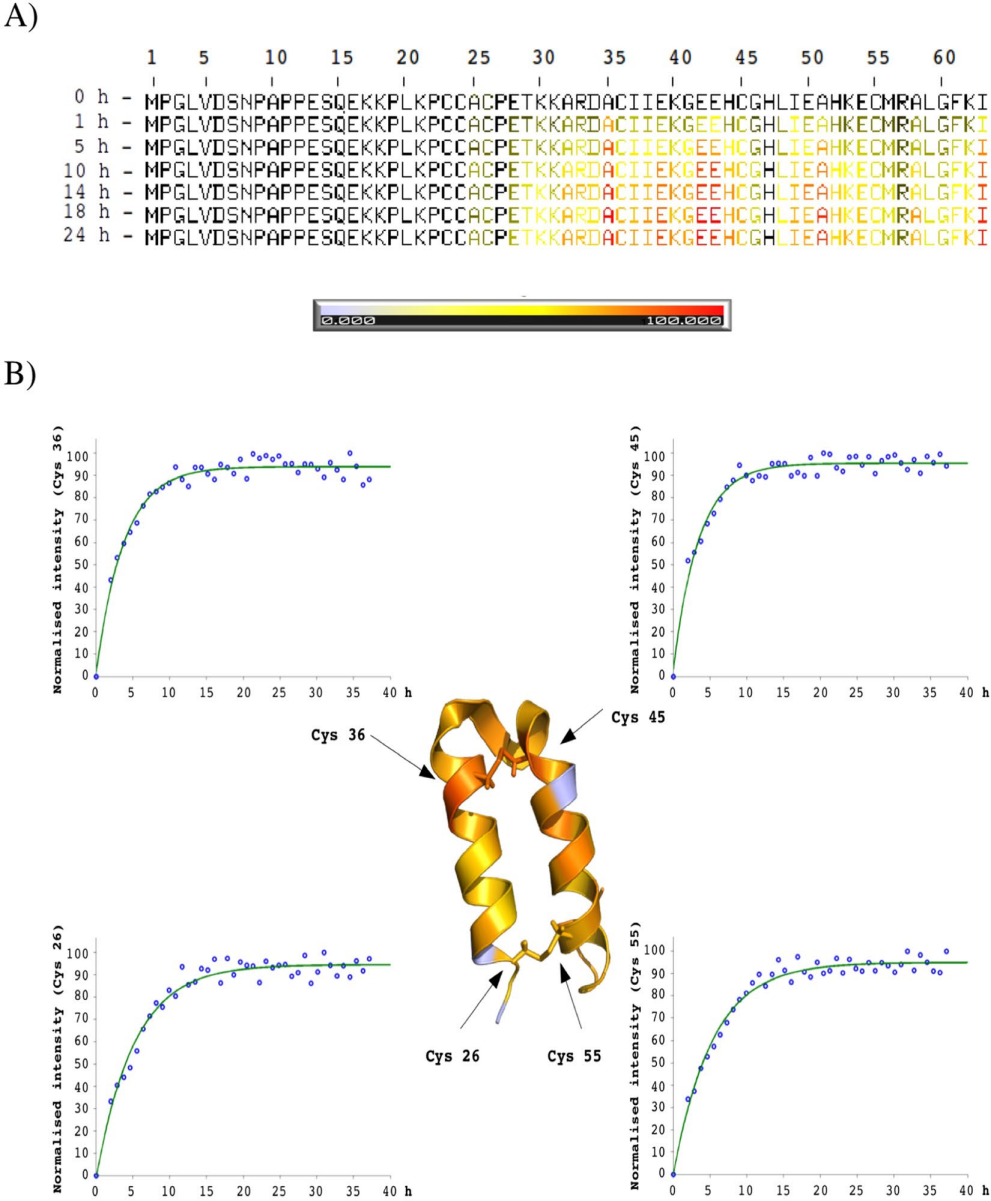
hCox17 oxidative folding followed by real-time solution NMR. Reduced hCox17 was allowed to refold and HSQCs were collected along the reaction. Residues were assigned and their intensities measured. (**A**) Normalized chemical shift intensities depicted according to the color bar, where in white is 0% formation and in red 100% formation. Residues 1 to 25 remained disordered and are not taken into account, His 47 was not assigned. (**B**) Normalized chemical shift intensities after 22 h of reaction shown on top of hCox17 solution NMR structure (PDB file: 2RN9), color code as in A. Folding kinetics from Cys 26, Cys 36, Cys 45 and Cys 55 amino acids are represented as an example. In the x axis time is represented as hours, and in y axis normalized chemical shift intensities.

Figure 8A shows the time evolution represented by a color scale with residue resolution. Under the refolding conditions most of the peaks adopted near final conformation before 22 h. The transition from the disordered to the folded state is nucleated by the formation of the inner disulfide bond (Cys36-Cys45), in agreement with previous results. Quite striking is the relative rapid folding of the residues involved in the stabilization of the α–hairpin fold, including a small hydrophobic core around the covalent link and Arg33 and Glu42 involved in a electrostatic interaction that connects helix I and the 3^10^ helix located between the two α–helical segments^17^. Residues around the second disulfide bond only display native like chemical shifts later on the reaction, whereas the N-terminal tail and Cys23 and Cys24 remain disordered along all the experiment. Figure 8B and S7 show the exponential changes in intensity, reflecting the differences in the progression of the folding reaction between residues.

### Mia40 accelerates the transition of hCox17 to the folded state acting on the inner disulfide bond

**Figure 9:**
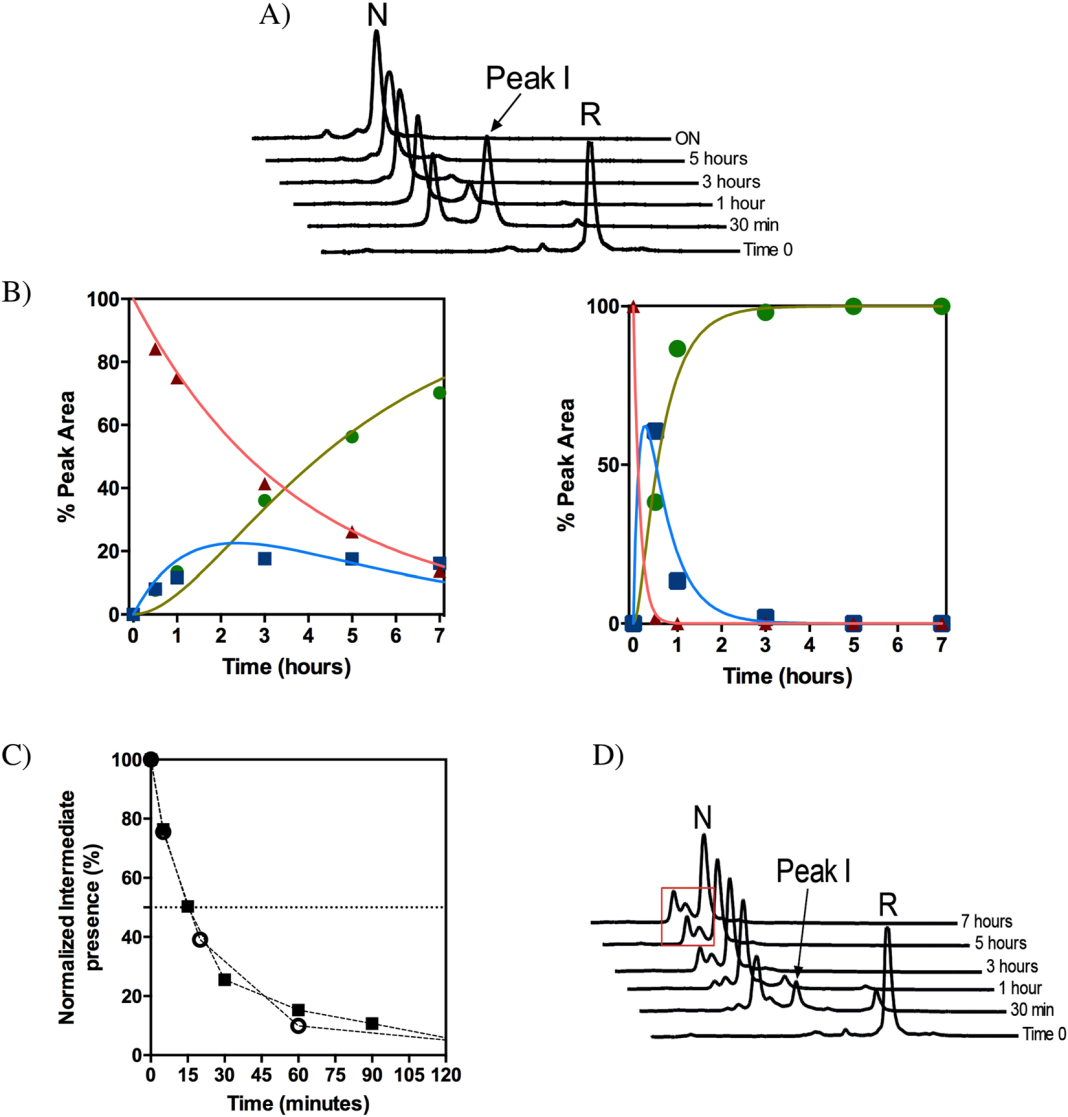
Impact of Mia40 and GSSG in the oxidative folding reaction of hCox17. (**A**) Reduced hCox17 was allowed to refold in the presence of Mia40 (molar ratio 1:2) and the reaction analyzed by RP-HPLC. (**B**) The normalized peak area of reduced (red triangles), intermediate (blue squares) and native (green circles) species are represented as a function of time in the presence (left) and absence (right) of Mia40. The fitting to a U-I-N transition is displayed in solid lines. (**C**) hCox17 folding intermediate disappearance as a function of time in the presence of Mia40 (open circles) and in stop and go assays (solid squares). A dashed line at 50% is also represented to identify the half-life of the specie. (**D**) RP-HPLC chromatograms of hCox17 folding reaction in the presence of 1 mM GSSG. Red boxes highlight off-pathway intermediates.

We proceeded analyzing the effect of Mia40 on the evolution of the different species identified in the hCox17 folding process using the approach we previously reported for Cox19^14^. Indeed, although the Mia40 pathway consists of at least two more components, Erv1 and cytochrome *c*, Mia40 has been shown to fully catalyze substrate oxidation when added in excess^12,38^. Mia40 is incorporated to the reaction as a fusion with GST which allows to dissect its impact on the hCox17 folding reaction by HPLC-RP since it does not elute within the used organic solvent gradient. When an excess of Mia40 was mixed with reduced hCox17 an overall acceleration of the folding reaction was observed (Fig. 9A). As it occurred for Cox19^14^, the folding pathway in the presence of Mia40 involves the same species as in non-catalyzed folding, the inner disulfide intermediate being formed rapidly in the first minutes, followed by the formation of native hCox17. Our results also suggest that the reaction can be separated in two independent steps and, accordingly, that Mia40 does not release the fully oxidized substrate, but the same semi-oxidized and compact inner disulfide bonded species that was observed during non-catalyzed folding. We tested this hypothesis using a simple kinetic model that considers the transition from unfolded (U) to a single intermediate (I) with a rate constant K_1_ and the latter one to the native protein (N) with a rate constant K_2_ and fitting the areas from the RP-HPLC curves of the species in the absence and presence of Mia40 (Fig. 9B). The r^2^ is >0.95 for all the species in both conditions. As we expected, K_1_ suffers a large 23-fold increase, from 0.3 to 6.9 h^−1^ in the presence of Mia40, whereas the change in K_2_ is much more moderate, a 2.5-fold increase, from 0.7 to 1.8 h^−1^. These data indicate that the formation of the inner disulfide is the rate-limiting step during non-catalyzed folding, whereas, in the presence of Mia40, it is the other way around. In addition, when there is no more reduced species available, the complete depletion of the intermediate requires the same time (∼2 hours) both in the absence and presence of Mia40 (Fig. 9C), which supports that, at least in our experimental setup, Mia40 promotes the transition of hCox17 towards the folded state mainly by accelerating the formation of the inner disulfide bond.

### The redox environment impacts both hCox17 folding rate and pathway

To test the influence of the redox environment in hCox17 folding we used oxidized glutathione (GSSG) as a thiol catalyst. GSSG strongly accelerates the conversion of reduced hCox17 towards the native species, with a rate that approaches that of Mia40 catalysed reaction (Fig. 9D). Despite GSSG can promote the formation of both native and non-native disulfide bonds in an unspecific manner, peak I continues being the major 1-SS species, which reflects a sequence encoded preference to form the inner disulfide bond. The acceleration of the reaction comes, however, at the cost of the accumulation of highly compact off-pathway isomers, according to their elution in RP-HPLC. These forms cannot evolve towards native hCox17, likely because the presence of GSSG impedes disulfide reshuffling and proofreading.

### Inducing secondary structure formation in the reduced state does not increase hCox17 folding rate

**Figure 10:**
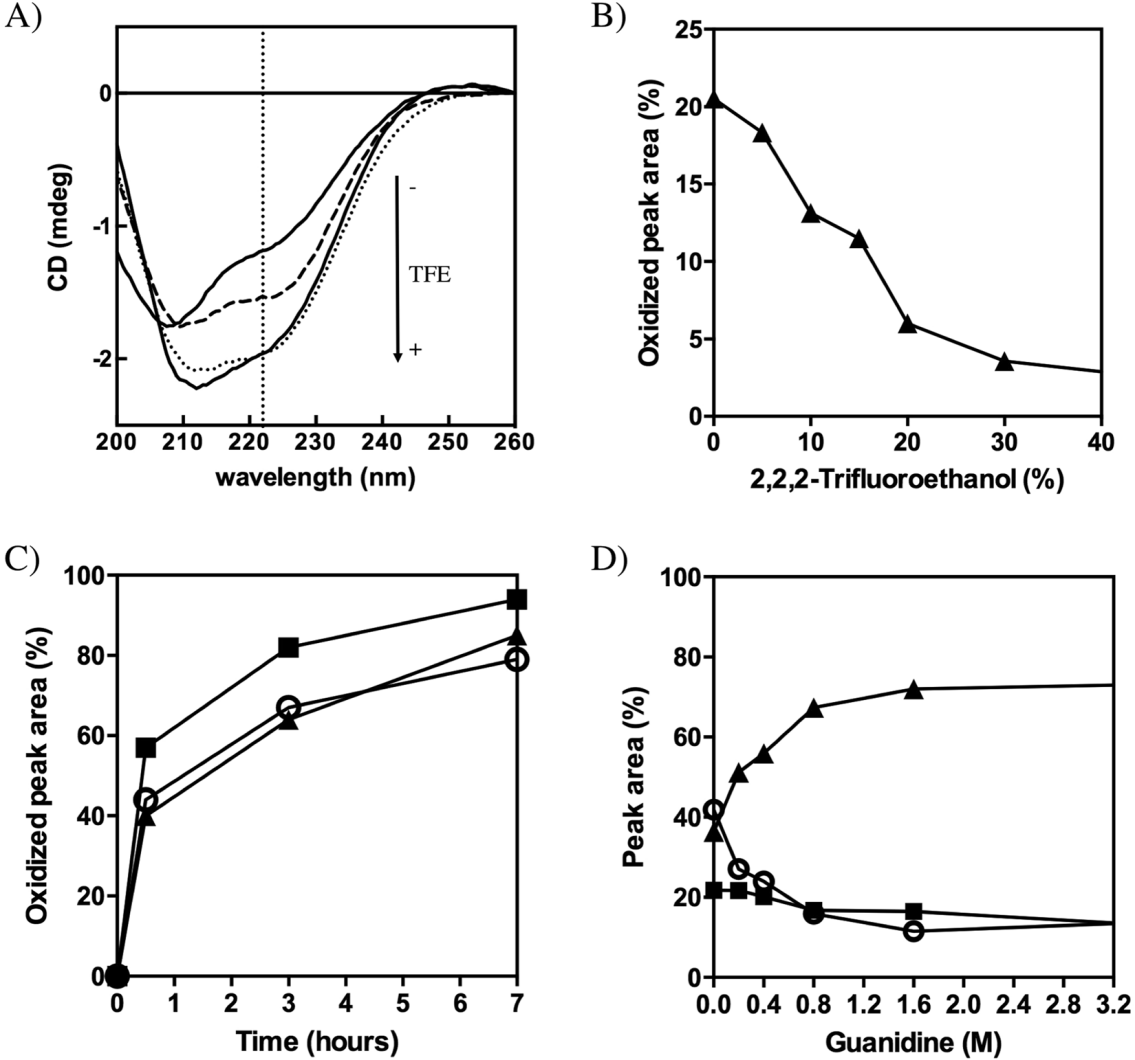
Influence of secondary structure in the oxidative folding of hCox17. (**A**) Far-UV CD spectra of reduced hCox17 in the absence (upper solid line) and in the presence of 10% (dashed line), 20% (dotted line) and 40% (lower solid line) of TFE. The characteristic α-helix 222 nm signal is indicated as a vertical dotted line. (**B**) Reduced hCox17 was allowed to refold in the presence of increasing concentrations of TFE. After 1 h of reaction, the samples were acid-quenched and analyzed by RP-HPLC. The native peak area was integrated and plotted as a function of TFE concentration. (**C**) Reduced hCox17 mutant H1 (circles), H2 (squares) and *wild type* (solid triangles) proteins were allowed to refold and the normalized native RP-HPLC peak areas are plotted as a function of the refolding time. (**D**) hCox17 refolding in the presence of increasing concentrations of Guanidine-HCl was quenched with TFA after 2 hours of reaction. The amount of native (solid triangles), intermediate (solid squares) and reduced (open circles) species are plotted as a function of denaturant concentration.

We tested whether induction of helical formation in the reduced state of hCox17 could accelerate the conformational transition towards the native state as we observed previously for Cox19 (14). The fluorinated alcohol 2, 2, 2-trifluoroethanol (TFE) promotes a concentration dependent coil-to-helix transition in peptides and disordered proteins^39^. Accordingly, addition of increasing concentrations of TFE, from 5 to 40%, while keeping the pH at 8.4, to reduced hCox17 resulted in an increase in its helical content, as deduced from the CD spectra (Fig. 10A). However, when we assessed the impact of TFE on hCox17 folding reaction we could not observe any increase in folding rates. Indeed, the folding rate is reduced as the helical content increases (Fig. 10B), a result that could respond to an inhibitory effect of pre-formed helical structure or alternatively TFE-induced formation of non-native helical conformations^40^.

The helix/coil transition algorithm AGADIR predicts very low propensities for both hCox17 helices, 2.27% for helix I and 0.47% for helix II (Table S4). We redesigned individually helices I and II to test whether an increase in their local α-helix propensities had any impact on the kinetics of hCox17 folding. To this aim we introduced residues with higher intrinsic helical propensity and novel i + 3, i + 4 contacts between side chains (Table S4), a strategy that has been used to drastically stabilize and increase the folding rates of helical proteins^41^. The expected increase in helical content upon mutation was 6-fold for helix I and 10-fold for helix II (Table S4). The two mutants were purified to homogeneity and their folding rates compared with WT hCox17. As shown in Fig. 10C, helix I mutant folds at the same rate that WT. helix II mutant folds faster than WT, but the impact is small, only 1.5-fold.

To further discard that residual helical propensity in the reduced state could play a relevant role in the folding of hCox17, we tested if the reaction could be completed in the presence of GdnHCl, a classic denaturant agent that will difficult the formation of secondary structure at the early stages of folding. At low concentrations (<3 M) the presence of GdnHCl does not preclude hCox17 folding, but instead accelerates the reaction in a concentration dependent manner, achieving maximum rates at 1.6 M concentration (Fig. 10D), which indeed suggests that transient interactions in the reduced state can play a certain inhibitory effect on hCox17 folding.

## Discussion

From as early as Anfinsen seminal work we know that the structure of globular proteins is imprinted in its genetic sequence^42^. It is also true that the lack of specific regular structure, characteristic of IDPs, is coded in the protein sequence^43,44^. The question that arises is: How can a sequence encode at the same time for ordered and disordered conformations when both states are biologically relevant? This is the case of hCox17, for which the initially disordered state is a requirement for its diffusion across the mitochondrial outer membrane, whereas the transition to a folded state in the IMS is necessary to transfer copper to cytochrome c oxidase. This places hCox17 in the new of class of conditionally disordered proteins, defined as polypeptides that under physiological conditions can exist in at least two states, one that shows a high degree of flexibility and disorder and a second state that shows a higher degree of order^21^. This mechanism allows to fine tune the function of these proteins according to the environmental context with a low energy cost. The slow folding reaction of the CHCH hCox17 domain and the possibility to trap and identify the major intermediates that populate the pathway has allowed us to integrate a set of techniques reporting in real time on covalent linkage, compactness, secondary structure and protein shape as it folds towards its functional conformation.

Disulfide bond formation is error-prone, particularly at the early stages of packing, often resulting in the accumulation of mispaired intermediates, which slows down folding reactions, since reshuffling is required to attain the native disulfide connectivity^45^. Despite being potentially able to form a large number of covalently-linked intermediates, hCox17 folds using sequential native disulfide formation, starting with the inner disulfide followed by the formation of the second outer disulfide, which results in a highly funneled pathway where a single native cross-linked on-pathway intermediate is accumulated, both in the absence and presence of Mia40. In the view of the simplicity of hCox17 folding pathway one would expect it to proceed fast. In contrast, spontaneous folding of hCox17 is exceedingly slow. The same behaviour was observed for Cox19, yet another substrate of Mia40 belonging to the CX_9_C family^14^. This parsimony allows enough residence in the reduced and disordered state for the proteins to be translocated from the cytosol to the IMS, since folding is a strong kinetic competitor of mitochondrial import^11^.

The succession of events leading to hCox17 folding is neither governed by the local vicinity of Cys residues, nor by the reduction in entropy of the system upon disulfide crosslinking, since the closure of the outer disulfide bond, or a non-native Cys23-Cys55 connection would close a much longer loop that the inner bond. This points at differential structural protection upon disulfide formation as the most probable factor responsible for the observed hCox17 folding hierarchy. It is clear from our data that the formation of the first disulfide bond is associated with the transition of hCox17 from an initially disordered state to a compact conformation. Structural collapse, disulfide formation and local secondary structure formation occur in a coordinated way, according to NMR, SAXS, FTIR and MS data. In excellent agreement with our results, Banci and coworkers, showed that a mimic of the inner disulfide bond intermediate, in which the outer Cys were mutated to Ser, exhibited stable inter-helical interactions and displayed a structure similar to that of native hCox17 containing two disulfides^46^. NMR data indicate that the first hCox17 regions to attain native chemical shifts are those around the inner disulfide where a small hydrophobic core and an electrostatic interaction between the carboxylate group of Glu42 and the guanidine group of Arg33 exist. These interhelical interactions surround the inner disulfide bond and would bring the two helices close to each other into the detected compact conformer. This species is likely a stable disulfide secure intermediate in which the disulfide bond is significantly protected from SH/SS reshuffling and reduction, and thereby is able to form the native protein directly, *i.e*., by oxidation of their exposed and reactive thiols. These properties suggest that this specie would not diffuse freely backward to the cytosol. The second disulfide can thus form more slowly in the IMS, without affecting much the equilibrium between unfolded and folded species.

It is generally assumed that protein folding drives subsequent disulfide formation, however our data suggest that conditionally disordered proteins like hCox17 deviate from this general rule, all the data converging to indicate that the formation of the inner disulfide is itself the molecular event triggering the transition of hCox17 between the initially disordered state and the folded functional conformation. This would promote immediate native-like compactness by forcing and stabilizing the interactions that envelop this covalent link. Indeed, NMR data indicate that all the residues between and adjacent to Cys36 and Cys45 arrive to their native positions with a similar fast speed, independently if they are polar, charged or hydrophobic. This occurs before the two α-helices have attained their definitive relative positions. In fact, NMR and SAXS data indicate that despite the inner disulfide intermediate has already attained a compactness equivalent to that of the native state, is still flexible. This might explain the accelerating role of glycerol, known to reduce protein flexibility^47^, which by decreasing the motion of the yet metastable helices might favor the formation of the outer disulfide bond.

In a way, the transition between the disordered and ordered states of hCox17 can be considered as a conformational switch controlled by a single selective post-translational modification: inner disulfide oxidation. Spontaneous formation of helical structure and hydrophobic collapse are usually fast events that cannot act as the drivers for the folding of a protein that should stay long in a disordered state waiting for translocation. Disulfide oxidation in the cytosol is a much slower reaction that might provide an effective time window for protein import. Another point to consider in hCox17 translocation to the IMS is aggregation. Indeed, the unfolded or partially unfolded states of globular proteins usually have an exacerbated propensity to aggregate and are recognized by chaperones to mediate their degradation^48^. How does hCox17 avoid these side reactions in the cytosol? An analysis of its sequence with the TANGO aggregation prediction algorithm indicates that it is absolutely devoid of aggregation-prone sequences (not shown), a property that shares with a number of bona fide IDPs^49^. This explains why the protein can remain soluble for long time in the unfolded state and why it lacks a defined nucleus that can trigger its folding. The disordered state of hCox17 should be viewed as an essential component of hCox17 function and therefore its sequence has evolved to optimize its properties as well as those of the folded state.

Many disordered proteins refold when they bind their partners and, indeed, this is the case of hCox17 *in vivo*, which folds in the IMS upon binding to Mia40. We show here that this binding event acts a catalyser of a disorder-to-order transition that is already delineated in the primary sequence of this conditionally disordered protein, since the detected folding intermediates in catalysed and non-catalysed reactions are identical. Indeed, unspecific oxidants like GSSG suffice to attain folding speeds equivalents to those obtained in the presence of the chaperone. However, this acceleration comes at the cost of the formation of off-pathway non-native linked intermediates, which indicates that Mia40 allows preserving the protein intrinsic propensities during accelerated folding, in good agreement with previous results obtained by Banci and co-workers, using Cys to Ser mutants of hCox17 and solution NMR^50^. The formation of the inner disulfide is the rate-limiting step in the folding reaction of hCox17 and therefore it prevents that hCox17 would fold prematurely. As expected for a canonical foldase, Mia40 catalyses this rate-limiting molecular event, having limited impact on the formation of the second native disulfide, at least in our experimental conditions.

There are two main models^51,52^ explaining how a disordered protein regains structure by binding to its partners: 1) The conformational selection model, which assumes that a small proportion of the intrinsically disordered polypeptide population is in appropriate metastable configuration, capable to interact with its target. 2) The folding upon binding model, which assumes that intrinsically disordered regions first bind to the partner to subsequently fold. The recent observation that in the disordered ensemble of yCox17, helix II seems to be transiently populated has suggested that Mia40 substrates from the CX_9_C family would fold according to a conformational selection mechanism, which would result in the initial formation of the second helix, oxidation of the inner disulfide and subsequent formation of the first helix and the outer disulfide^53^. The sequence of helix II in yCox17 has much higher intrinsic helical propensity (18.78%) than any of the two helices in hCox17 and our data does not support such conformational selection driving the folding of hCox17. Indeed, it has been just proposed that Mia40 acts as a holding trap, trapping incoming disordered substrates via hydrophobic binding, a reaction that is essential and sufficient for translocation, immediately followed by catalyzed oxidative folding^54^, a pathway that is consistent with a folding upon binding mechanism. The oxidoreductase and holdase activities of Mia40 seem to reside in different regions of the protein and are both essential. However, despite an oxidase-deficient Mia40 mutant is inviable, it can be partially rescued by the addition of exogenous chemical oxidants^54^, an observation that suggests that many Mia40 substrates encode for its spontaneous transition between a disordered translocation-competent state and its functional conformation, as we show here for hCox17.

## Materials and Methods

### Analysis of protein disorder

Intrinsic disorder in hCox17 was predicted using the web-based algorithms FoldIndex^24^, IUPRED^26^, ESpritz^25^, PONDR-FIT^27^ and RONN^28^ with default settings. An 11-residue sliding window was used in all cases.

### Protein purification

hCox17-GST cDNA, gently sent by L. Banci and coworkers, was transformed to *E. coli* BL21 DE3 and *E. coli* Origami2 cells in order to express *wild type* (WT) and mutant proteins. As the protein did not have any Trp or Tyr residue in its sequence, a point S7 W mutation was introduced in the disordered N-tail. This mutation did not affect the kinetics or the species population during the oxidative folding and allowed us proper quantification in the different assays. The protein was expressed and purified as previously described^17^.

### Infrared spectroscopy

FTIR spectra of human hCox17 were measured at 6 mg/mL in 50 mM Tris pH 8.4, 100 mM NaCl in D_2_O. Measurements were performed on a FT600 Bio-Rad spectrometer equipped with a MCT detector, using a demountable liquid cell with calcium fluoride windows and 50 μm spacers. Typically, 500 scans for each background and sample were collected and the spectra were obtained with a nominal resolution of 2 cm^−1^ at 22 °C. The spectra were acquired in slow kinetics mode and the measurements were performed continuously for 6 h. The buffer contribution to the spectra was subtracted. Data treatment and band decomposition of the original amide I’ band was performed as previously described with GRAMS 9.0 software^55^. For each component, four parameters were considered: band position, band height, band width, and band shape. In decomposing the amide I’ band, gaussian components were used. The number and position of the component bands were obtained through Fourier self-deconvolution using Lorentzian lineshape and the parameters recommended for protein analysis, 13 cm^−1^ for full-width at half-height (FWHH) and 2.4 for the resolution enhancement factor^55^. Initial heights were set at 90% of those of the original spectrum for the bands in the wings and for the most intense component and at 70% of the original intensity for the other bands^55^. The curve-fitting procedure was accomplished in two steps: i) The band position was fixed, allowing width and heights to approach final values, and ii) band positions were left to change. The quality of the fitting was evaluated visually by overlapping the reconstituted overall curve on the original spectrum and by examining the residual obtained by subtracting the fitting from the original curve. Correlation coefficients between the original and the fitted spectra were ≥0.999. The mathematical solution of the decomposition may not be unique, but if restrictions are imposed, such as maintenance of the initial band positions in an interval of ±1 cm^−1^, preservation of the bandwidth within the expected limits, or agreement with theoretical boundaries or predictions, the result becomes, in practice, unique.

### Size Exclusion Chromatography

Oxidized and reduced hCox17 were injected into a Superdex 75 100/30G GL column (GE Healthcare) coupled to an AKTA - Fast Protein Liquid Chromatography device (GE Healtcare), previously equilibrated with 50 mM Tris pH 8.4, 100 mM NaCl buffer. hCox17 elution was monitored by following absorbance at 280 and 214 nm. Initial protein concentration was 50 mM.

### CD spectroscopy

Far UV CD spectra were acquired on a Jasco-710 spectropolarimeter continuously purged with nitrogen and thermostated at 25 °C and quartz cuvettes of 2 mm width. Spectra were recorded from 260 to 200 nm setting the bandwidth at 1 nm, scan speed at 200 nm/min and data pitch at intervals of 0.5 nm. For folding experiments, fully reduced hCox17 with 200 mM DTT overnight was allowed to oxidize in 50 mM NaCl, 20 mM phosphate buffer at pH 7.0 after removing the reducing agent with a PD-10 desalting column (GE Healthcare, REF. 17-0851-01). Scans with a data pitch of 0.5 nm from 200 to 260 nm were collected every 30 minutes for 20 hours. For thermal denaturation studies, the molar ellipticity at 222 nm was recorded each 0.2 °C with an increasing heat rate of 1 °C/min in the 25 to 95 °C range. For TFE experiments, reduced hCox17 was mixed with TFE at 10, 20 and 40% final concentration in 20 mM phosphate buffer 50 mM NaCl and the pH adjusted to 7.0.

### High-Performance Liquid Chromatography

hCox17 at 1 mg/mL was reduced in 50 mM Tris pH 8.4, NaCl 100 mM and 200 mM DTT over night at room temperature. To initiate folding, samples were loaded in a PD-10 desalting column previously equilibrated with 50 mM Tris pH 8.4, NaCl 100 mM. Selected concentrations of GSH, Glycerol, TFE and Mia40 were added to the collected fraction to modulate or enhance the folding kinetics. To follow the oxidative reaction, removed aliquots at selected times were quenched with 2% TFA and loaded in a *Waters 2690 HPLC* system with a C4 column *(Phenomenex) 250* × *4.6 5* *mm*. A linear gradient of 20–35% of 0.1% TFA in acetonitrile was applied for 60 minutes at a flow-rate of 0.75 ml/min, Eluted species were monitored by a coupled *Waters 2487* detector at 214 nm and 280 nm wavelength. In the same way, a preparative *VYDAC protein C4* was used to purify the intermediate species of the reaction. An increased 3 mL/min flow was applied to the same linear gradient of acetonitrile as above for 120 minutes. The purified species were identified by mass spectrometry and the reaction intermediate were quantified for the stop/go experiments in 50 mM Tris pH 8.4 NaCl 100 mM at 0.5 mg/mL^14,22^.

### MALDI-TOF and TOF/TOF analysis

The assays were performed on an Autoflex Speed mass spectrometer (Bruker, Bremen). The free Cys residues of Peak I folding intermediate species were alkylated by adding the samples, dissolved in 0.1% trifluoroacetic acid (30 μL), to an excess of 75 mM iodoacetamide in 50 mM ammonium bicarbonate buffer, pH = 8, (70 μL), enough to rise the pH of the reaction mixture, to minimize the risk of disulfide reshuffling. The reaction was allowed to proceed for 30 min at room temperature.

Samples before and after alkylation were then analyzed by MALDI-TOF mass spectrometry using sinapinic acid as matrix. A mixture of protein standards was used for internal calibration.

For fingerprint analysis, the alkylated proteins were first purified using ZipTip (Millipore) C18 micro columns, evaporated, redissolved in 1 M Urea, 25 mM ammonium bicarbonate, subjected or not to reduction by treatment with 10 mM DTT for 1 hour at r.t., and digested with trypsin (3 h at 37 °C). The digests were then purified using ZipTip C18 and analyzed by MALDI TOF/TOF mass spectrometry using HCCA as matrix.

### Electrospray-ionization mass spectrometry (ESI-MS)

Lyophilized hCox17 (0.5 mg) was dissolved in 3 mL of 10 mM ammonium acetate, pH 8.0, 50 mM DTT, and incubated statically overnight at room temperature, in order to reduce Cys residues. The sample was buffer-exchanged by reversed-phase chromatography (C-18 Zip Tip, Merck-Millipore, Darmstadt, Germany), eluting by 50% acetonitrile, 0.5% formic acid, and dried in a Speed Vac centrifugal system (Analitica De Mori, Milano, Italy). The pellet was ressuspended in 3 mL of 10 mM ammonium acetate, pH 6.7, to initiate oxidative refolding and incubated statically at room temperature for variable time. Aliquots were injected into a hybrid quadrupole-time of flight (TOF) mass spectrometer (QSTAR Elite, AB Sciex, Forster City, CA, USA), employing metal-coated borosilicate capillaries with emitter tip of ~1 µm internal diameter (ThermoFisher Scientific, Waltham, MA, USA). The main instrumental parameters were: ion-spray voltage, 1.1 kV; curtain gas, 20 PSI; declustering potential, 80 V. The sample source and the instrument interface were kept at room temperature. Spectra were averaged over 1-minute acquisition.

### Small-angle X-ray scattering

SAXS measurements were carried out on ESRF beamline BM29 at λ = 0.992 Å^56^. Scattering curves were recorded in the momentum transfer range 0.033 < *s* < 0.498 Å − 1 (*s* = 4π sin (θ)/λ, 2θ is the scattering angle) using a Pilatus 1 M detector (Dectris Ltd., Baden, Switzerland). Two samples of hCox17 at 6 mg/mL and 4 mg/mL were reduced with DTT, then the reducing agent was removed as described for oxidative folding assays in HPLC to give rise to final concentrations of 3 mg/mL and 2 mg/mL in a 50 mM Tris pH 8.4 NaCl 100 mM buffer. Both solutions were allowed to air oxidize and small volumes (40 μL) were taken periodically during 10 (2 mg/ml) and 12 (3 mg/mL) hours and submitted to a SAXS measurement. Buffer samples were measured before and after protein solutions. Data reduction, correction for radiation damage, and buffer subtraction, were performed by the beam line automated procedure. Both concentrations provided equivalent curves with to indication of interparticle interactions and were treated independently. The radius of gyration, *R*_*g*_, for each of the curves was derived using Guinier’s approximation with the ATSAS software^57^. The maximum particle size (*D*_*max*_) was determined from the pair-wise distance distribution function, *P(r)*, computed with PRIMUS^58^.

### Nuclear Magnetic Resonance

^15^N-labelled hCox17 was reduced in 50 mM Tris pH 8.0, 100 mM NaCl and 100 mM DTT overnight at room temperature. To initiate the folding process, the sample buffer was exchanged to 50 mM Tris pH 6.5, 100 mM NaCl and 1 mM DTT using disposable PD-10 desalting columns. Subsequently, the protein was concentrated up to 150 μM. Refolding process was monitored by collecting ^1^H-^15^N HSQC spectra every 52 minutes for 36 hours (starting at 2 h) in an 800 MHz Bruker Avance III spectrometer at 298 K. Resonances were assigned based on Banci’s previous work (BMRB: 11019 & 11020)^17^. Peak intensities were measured using NMRPipe^59^. Normalized folding transition percentage for each residue in hCox17 was calculated using in-house built MatLab^®^ scripts.

^15^N-^13^C-labelled hCox17 was reduced in 50 mM Tris pH, 100 mM NaCl and 100 mM DTT. ^1^H-^15^N-HSQC and HNCACB spectra were collected in an 800 MHz Bruker Avance III spectrometer at 298 K. Backbone chemical shifts were assigned for Ala, Trp and Ser and their neighbouring residues as they have a clear Cβ chemical shifts. Protein flexibility and secondary structure was predicted by comparing the Random Coil index values from the backbone chemical shifts (HN, N, Cα and Cβ)^31^.

Backbone amide ^15^N relaxation data (R_1_, R_1ρ_ and heteronuclear ^15^N-^1^H-NOE were measured for hCox17 at 600 MHz by published methods^60^. For R_1ρ_ experiments, the employed spin-lock field was 3 kHz. Sampled relaxation time points ranged from 0.1 to 1.2 s for R_1_, and 50 to 250 ms for R_1ρ_. For the heteronuclear ^15^N-^1^H-NOE experiment, steady-state HN saturation was achieved with a 4 s train of square 120° pulses, and a 12 s interscan delay was used in the reference (non-saturated) spectrum. The backbone ^15^N relaxation data was analyzed with the Lipari-Szabo formalism^61^ using in-house MatLab^®^ scripts.

## Electronic supplementary material

Supplementary information
