# Supplementary material for: Disulfide driven folding for a conditionally disordered protein
Source: Sci Rep. 2017 Dec 5;7:16994. doi: 10.1038/s41598-017-17259-4 (PMC5717278; doi:10.1038/s41598-017-17259-4)
Supplement: Supplementary file 1 — Supplementary information [file 41598_2017_17259_MOESM1_ESM.pdf]

Supplementary Material for:

Disulfide driven folding for a conditionally disordered protein

Hugo Fraga[1,2,3‡], Jordi Pujols[1,2‡], Marcos Gil-Garcia[1,2], Alicia Roque[2], Ganeko Bernardo-Seisdedos[4], Carlo Santambogrio[5], Joan-Josep Bech-Serra[6], Francesc Canals[6], Pau Bernadó[7], Rita Grandori[5], Oscar Millet[4] and Salvador Ventura [1,2]*

[1] Institut de Biotecnologia i Biomedicina. Universitat Autònoma de Barcelona, 08193-Bellaterra, Spain.

[2] Departament de Bioquímica i Biologia Molecular. Universitat Autònoma de Barcelona, 08193-Bellaterra, Spain.

[3] Departamento de Bioquimica, Faculdade de Medicina da Universidade do Porto, Portugal

[4] Protein Stability and Inherited Diseases Laboratory, CIC bioGUNE, 48160-Derio, Spain.

[5] Department of Biotechnology and Biosciences, University of Milano-Bicocca, Italy

[6] Vall d'Hebron Institute of Oncology (VHIO), Barcelona, Spain

[7] Centre de Biochimie Structurale, INSERM-U1054, CNRS UMR-5048,Université de Montpellier. 29, rue de Navacelles, 34090-Montpellier, France

‡ These authors contributed equally.

* Correspondence: salvador.ventura@uab.es; Tel.: (+34) 93 586 8956

Table S1. Reference chemical shifts. Chemical shifts for Ala, Trp and Ser and their neighbouring residues. Same nuclei chemical shifts are compared with BMRB 11019.

		COX17_Red				COX17_2S-S (BMRB 11019)			
Num.	Res.	N	H	CA	CB	N	H	CA	CB
-1	F			58,14	40,14				
0	T	117,71	8,36	61,91	70,19	118,31	8,32	60,2	72,47
1	M	124,95	8,59	53,67	32,95	125,37	8,53	51,88	34,59
9	P			63,14	32,34			61,49	34,1
10	A	126,11	8,49	50,58	18,46	126,75	8,56	48,93	19,95
13	E			57,26	30,38			55,52	32
14	S	117,1	8,61	64,2	58,7	117,43	8,53	56,87	65,84
15	Q	122,98	8,68	56,25	29,76	123,15	8,55	54,36	31,34
24	C			57,53	30,97			56,56	30,62
25	A	124,95	8,46	53,38	19,2	126,03	7,96	50,66	22,29
26	C	118,87	8,59	57,24	30,31	116,96	9,38	57,22	45,55
28	E			57,31	30,54			58,83	30,77
29	T	116,43	8,45	62,52	70,06	113,77	8,9	63,09	68,82
30	K	124,95	8,59	53,67	32,95	128,05	8,25	59,2	35,09
31	K			55,48	33,27			57,87	34,4
32	A	126,25	8,63	53,02	19,63	120,87	7,75	53,36	20,32
33	R	120,97	8,62	56,88	27,98	119,54	8,11	58,18	32,1
34	D			54,9	41,48			56,14	41,04
35	A	124,52	8,45	53,16	19,59	121,73	8,24	53,41	20,12
36	C	118,69	8,57	59,15	28,18	118,58	7,61	59,17	41,46
50	E			58,59	28,67			57,54	30,95
51	A	127,22	8,7	53,04	19,72	121,67	8,09	53,67	19,78
52	H	126,12	8,59	56,67	33,43	119,04	7,89	58,16	33,37
57	R			57,54	20,31			57,86	32,24
58	A	124,9	8,54	52,66	19,31	123,38	8,1	53,16	19,76
59	L	120,83	8,29	55,72	42,74	117,6	7,45	53,42	45,1

Table S2. FTIR contributions and assignments. Secondary structure assignments for selected time-points of a hCox17 refolding reaction at pH 8.4.

Assignment	0h		2.5h		6h	
	Band (cm^{-1})	%	Band (cm^{-1})	%	Band (cm^{-1})	%
**Turns**	1670.2	9	1671.3	7	1671	8
**Turns**	1660.2	5				
**α-helix**			1653.46	25	1653.46	44
**Random coil/flexible regions**	1642.8	86	1639.5	68	1640	48

Table S3. Disulfide pairing of hCox17 folding intermediate. Acid-trapped intermediate I was purified by RP-HPLC and alkylated by the addition of an excess of iodoacetamide solution in 50 mM ammonium bicarbonate buffer, pH 8. The alkylated intermediate was digested with trypsin with (+DTT) or without (-DTT) a previous reduction with DTT. MALDI-TOF mass spectrometry was used to analyse the tryptic digestion and identify each single resulting peptide; however, only those peptides providing evidence for the disulfide pairing assignment, are indicated. The identity of the identified peptides was further confirmed by TOF/TOF fragmentation analysis. While the treatment with iodoacetamide allowed the detection of free cysteines, the incubation with DTT allowed the detection of disulfide bridges. In the lower panel, we show a sequence map of the peptides used for disulfide identification in Peak I. In red cysteines that reacted with iodoacetamide, not forming disulfides in the intermediate, and in green cysteines in disulfide bonds (identified only after incubation with DTT). The only possibility is the presence of a Cys36-Cy45 disulfide bond.

**Peptide Sequence**	**$[M+H]^+$ Obs.**	**$[M+H]^+$ Calc.**	**-DTT**	**+DTT**	**Cys in S-S bonds**	**Free Cysteines (CAM reactive)**	**Cys-Cys**
[18-30] KPLKPC(CAM)C(CAM)AC(CAM)PETK	1588.81	1588.76	Y	N		23,24,26	
[18-30] KPLKPC(CAM)C(CAM)AC(CAM)PETK	1588.81	1588.76	N	Y		23,24,26	
[41-57] GEEHCGHLIEAHKEC(CAM)MR	2035.99	2035.88	N	Y	45	55	
[34-57] DACIIEKGEEHCGHLIEAHKEC(CAM)MR	2808.43	2808.26	N	Y	36,45	55	
[34-57] DACIIEKGEEHCGHLIEAHKEC(CAM)MR	2806.39	2806.25	Y	N		55	36-45
[34-53] DACIIEKGEEHCGHLIEAHK	2232.18	2232.05	N	Y	36,45		
[34-53] DACIIEKGEEHCGHLIEAHK	2230.13	2230.03	Y	N			36-45

**Peptide Sequence**	**MPGLVDWNPAPPESQEKKPLKPCCACPETKKARDACIIEKGEEHCGHLIEAHKECMRALGFKI**
[18-30]	**KPLKPCCACPETK**
[41-57]	**GEEHCGHLIEAHKECMR**
[34-57]	**DACIIEKGEEHCGHLIEAHKECMR**
[34-53]	**DACIIEKGEEHCGHLIEAHK**

Table S4. Sequence of the helix-designed mutants and their specific values of helical propensity according to AGADIR.

**Helix 1**	**Sequence**	**helix propensity (%)**
Wild Type	ACPETKKARDACIIEKGEE	2.27
Mutant H1	ACPE**E**KK**K**RDACI**LK**KGEE	13.63
**Helix 2**	**Sequence**	**helix propensity (%)**
Wild Type	HCGHLIEAHKECMRALG	0.47
Mutant H2	HCG**E**LI**QKY**KECMRALG	4.80

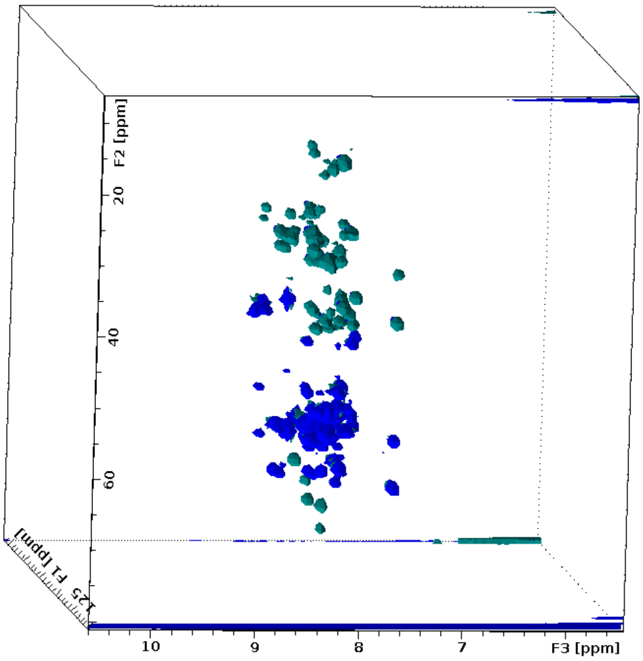

Figure S1. 3D cube representation of the HNCACB spectrum. Cα chemical shifts are represented in blue and Cβ are represented in green. Ala (15-25 ppm), Ser and Thr (55-75 ppm) are easily distinguished as shown in the image.

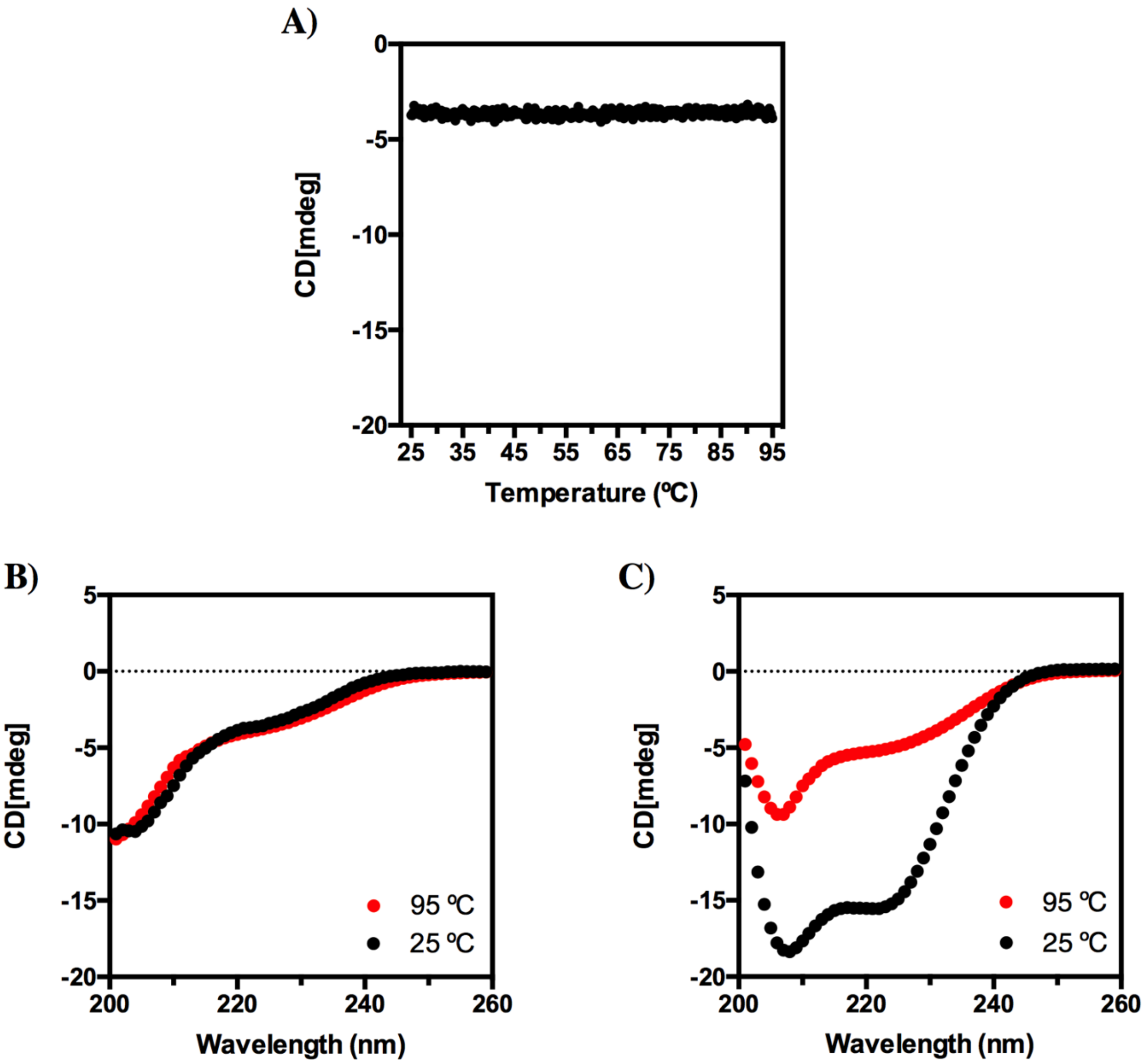

Figure S2. Thermal denaturation of hCox17. A) Thermal denaturation of reduced hCox17 followed by far-UV CD at 222nm. **B)** and **C)** Far-UV CD representative scans of reduced (**B**) and native (**C**) hCox17 incubated at 25ºC and 95ºC.

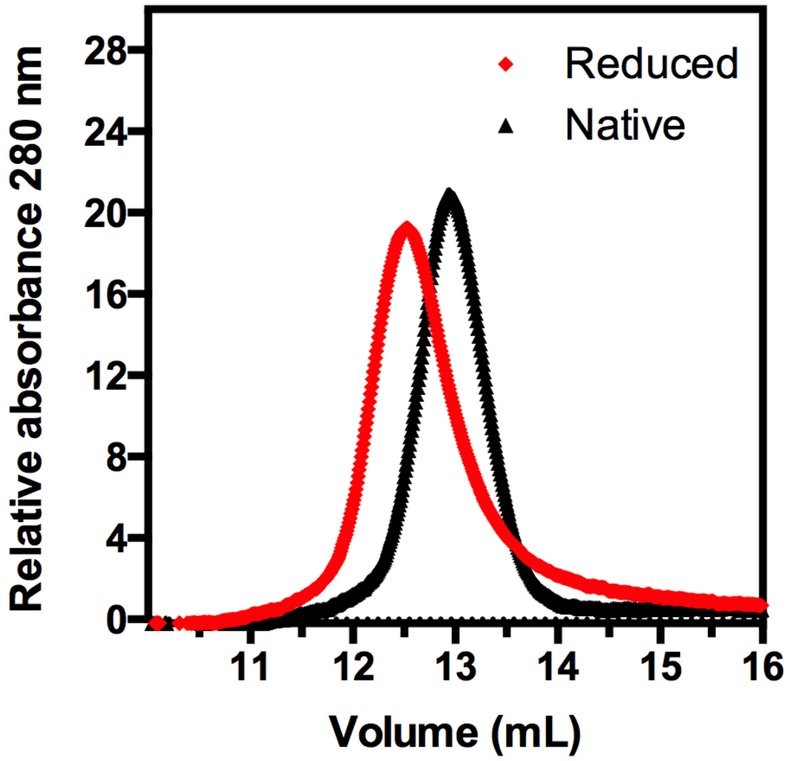

Figure S3. Hydrodynamic properties of hCox17. Reduced (red) and native (black) hCox17 were analysed by size exclusion chromatography (SEC) on a Superdex 75 column. Protein elution was monitored measuring absorbance at 280 nm.

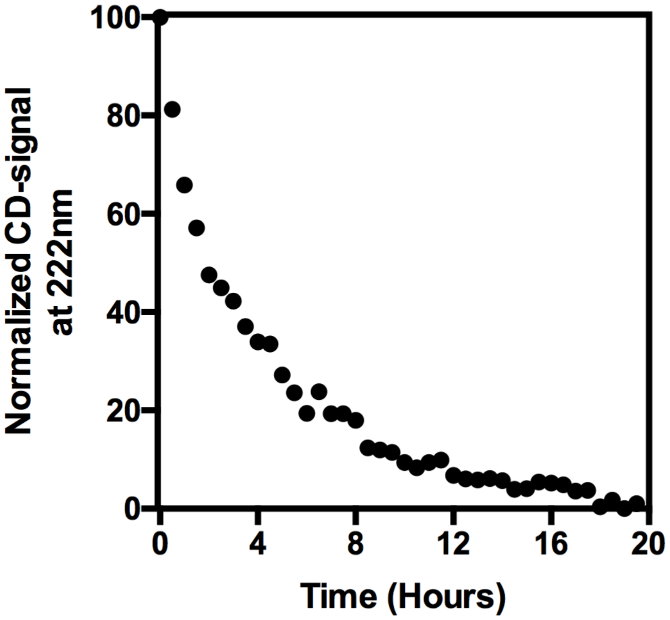

Figure S4. Refolding reaction of hCox17 in far-UV CD. Reduced hCox17 was allowed to refold at pH 7.0 inside a sealed CD cuvette and secondary structure formation was monitored using far-UV CD. Normalized signal at 222 nm is plotted as a function of time.

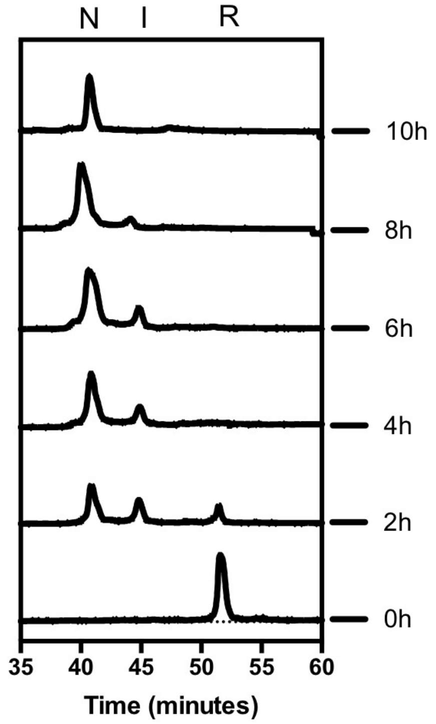

Figure S5. RP-HPLC analysis of a hCox17 refolding reaction at SAXS refolding conditions. Several RP-HPLC chromatograms corresponding to specific reaction times of hCox17 oxidative refolding at 3 mg/mL in Tris buffer pH 8.4.

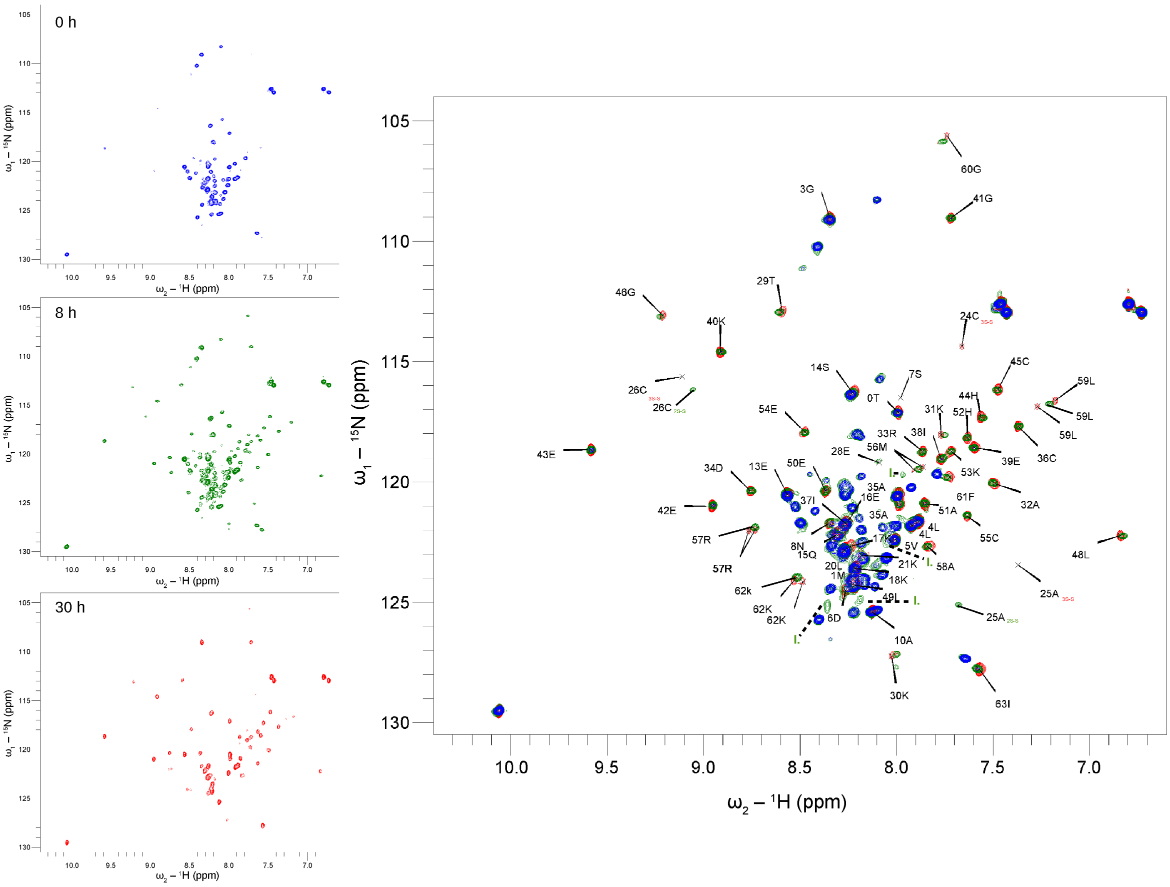

Figure S6. 1H-15N HSQC spectra of hCox17 folding process at different times. On the left different spectrum are shown, at 0 h (blue) at 8 h (green) and at 30 h (red). The central panel is an overlay of the three spectra. Chemical shifts are assigned for the N state of hCox17 protein. Some intermediate signals are indicated by green dashed lines (I).

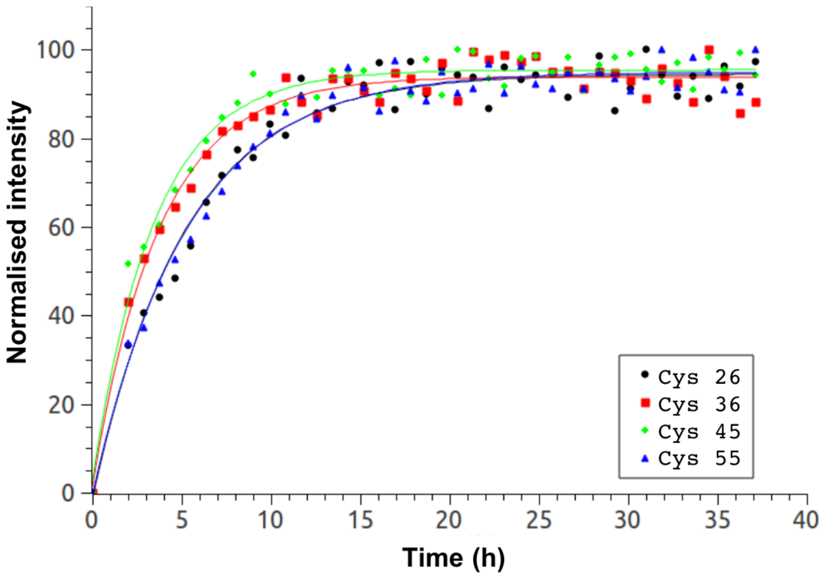

Figure S7. Reduced hCox17 was allowed to refold and HSQCs were collected along the reaction. Normalized chemical shift intensities of cysteines involved in hCox17 structural disulfides was plotted as a function of time.
